# The impact of mindfulness apps on psychological processes of change: a systematic review

**DOI:** 10.1038/s44184-023-00048-5

**Published:** 2024-03-18

**Authors:** Natalia Macrynikola, Zareen Mir, Tishmattie Gopal, Erica Rodriguez, Sunnie Li, Milann Cox, Gloria Yeh, John Torous

**Affiliations:** 1 Beth Israel Deaconess Medical Center & Harvard Medical School, Boston, MA USA; 2 Teacher’s College, Columbia University, New York, NY USA; 3 Hunter College, City University of New York, New York, NY USA; 4 Northeastern University, Boston, MA USA; 5 Penn State College of Medicine, Hershey, PA USA

**Keywords:** Psychology, Health care

## Abstract

Mindfulness-based interventions (MBIs) have demonstrated therapeutic efficacy for various psychological conditions, and smartphone apps that facilitate mindfulness practice can enhance the reach and impact of MBIs. The goal of this review was to summarize the published evidence on the impact of mindfulness apps on the psychological processes known to mediate transdiagnostic symptom reduction after mindfulness practice. A literature search from January 1, 1993, to August 7, 2023 was conducted on three databases, and 28 randomized controlled trials involving 5963 adults were included. Across these 28 studies, 67 outcome comparisons were made between a mindfulness app group and a control group. Between-group effects tended to favor the mindfulness app group over the control group in three psychological process domains: repetitive negative thinking, attention regulation, and decentering/defusion. Findings were mixed in other domains (i.e., awareness, nonreactivity, non-judgment, positive affect, and acceptance). The range of populations examined, methodological concerns across studies, and problems with sustained app engagement likely contributed to mixed findings. However, effect sizes tended to be moderate to large when effects were found, and gains tended to persist at follow-up assessments two to six months later. More research is needed to better understand the impact of these apps on psychological processes of change. Clinicians interested in integrating apps into care should consider app-related factors beyond evidence of a clinical foundation and use app databases to identify suitable apps for their patients, as highlighted at the end of this review.

## Introduction

Mindfulness-based interventions (MBIs) have demonstrated efficacy in improving a range of clinical outcomes, such as depression and anxiety^1^. In a rigorous randomized controlled trial, mindfulness-based stress reduction (MBSR) was even found to be non-inferior to antidepressant medication^2^. However, MBI delivery and impact remain limited by various factors, two important ones being barriers to access and difficulties with sustained engagement. That is, for many individuals, MBIs remain inaccessible for the same reasons that mental health treatment remains inaccessible, including cost, stigma, a shortage of clinicians, and various logistical barriers (e.g., lack of transportation, lack of childcare)^3,4^. In addition, MBIs necessitate practice outside of session, which contributes to outcomes^5^; however, many struggle to sustain a consistent mindfulness practice on their own outside of in-person sessions.

Technology can bridge the gap in both of these situations. Mindfulness apps can provide an alternative when in-person MBIs are inaccessible, and integrating mindfulness apps into in-person treatment can facilitate practice and increase intervention impact^6,7^. Yet most commercially available mindfulness apps have not been scientifically evaluated^8^, and most mental health apps struggle to keep users engaged^9^. Related, uptake of mindfulness apps is low in treatment, despite interest from clinicians^10^ and their patients^11,12^. One commonly cited barrier is a lack of knowledge about which apps are credible and effective^13^. To address these barriers and stimulate more research into building the mindfulness app evidence base, we conducted a systematic review to assess these apps’ effectiveness in shifting psychological processes of change related to mindfulness.

Recent reviews suggest that mindfulness app effects on clinical outcomes are often inconsistent. For example, one review found generally small app effects on depression and contradictory results for anxiety^14^. However, the common approach of evaluating app effects on such distal psychological outcomes as psychiatric disorders is problematic because app intervention periods tend to be too brief for these types of outcomes to demonstrate significant and consistent change. A recent meta-analysis of 23 mindfulness app evaluations found that only nine studies used intervention periods that adhered to the recommended eight weeks of such MBIs as MBSR and MBCT^15^. Therefore, a more suitable approach to reviewing mindfulness app efficacy may be to focus on the more proximal processes of change, or mechanisms, that have been empirically demonstrated to explain the effects of mindfulness practice on more distal psychological outcomes. Temporally, mechanisms shift first^16^; thus, focusing on these intermediary outcomes may provide a clearer picture of the efficacy of mindfulness apps.

Adopting a mechanisms-as-outcomes approach has three additional benefits. First, the knowledge gained from such an approach can lead to more targeted apps, which may enhance their efficacy. Second, current evidence suggests that mHealth app engagement in the general public falls to near zero after two weeks^9^. Given this reality, it is key to understand whether the brief periods in which apps tend to be evaluated have any impact on mechanistic targets. If they do not, it will be important to focus efforts on sustaining engagement for longer in the hopes of seeing a substantial impact on these important targets. Third, this approach provides valuable insights for clinicians specializing in evidence-based treatments as many of the mechanisms of mindfulness practice (e.g., emotion regulation) are also the transdiagnostic mechanisms targeted in such therapies^17,18^. Therefore, knowledge gained from this approach can aid clinicians in evaluating such apps as potential complements to ongoing treatment goals.

To date, no mindfulness app review of which we are aware has focused on the mechanisms of mindfulness training as outcomes. Thus, a systematic review is warranted to investigate the evidence of mindfulness app effects on the mechanistic processes through which mindfulness training has been demonstrated to influence transdiagnostic symptom change^19^.

## Methods

This systematic review was conducted according to PRISMA guidelines^20^ and registered on the International Platform of Registered Systematic Review and Meta-Analysis Protocols (#202350017). To identify mechanisms of mindfulness practice, we first searched for papers that proposed likely mechanisms based on a thorough rationale. We searched for these papers in Pubmed (using the keywords “mindful*” in the Title field, and “mechanism” or “mediat*” in the Text field). This method yielded four theory papers^21–24^, from which we extracted the proposed mechanisms. For each proposed mechanism, we then searched the literature for empirical support (obtained through mediation analysis). Our list of theoretically and empirically supported mechanisms of mindfulness practice appears in Table 1. (For an overview of corresponding theories, see eTable 1).

**Table 1:** List of outcomes of interest.

Mindfulness mechanism	Theoretical paper proposed in				Empirically supported?
	Shapiro	Holzel	MAT	MMT	
Decentering/defusion	x			x	yes
Self-regulation	x				yes
Values clarification	x				yes
Acceptance/psychological flexibility	x		x		yes
Awareness	x^a^	x^a^			yes
Nonreactivity	x^a^	x^b^			yes
Attention regulation		x	x		yes
Emotion regulation		x			yes
- Reappraisal		x^b^		x	yes
- Extinction		x^b^			yes
- Suppression		x^b^			yes
- Worry		x^c^			yes
- Rumination		x^c^			yes
Non-judgment		x	x		yes
Positive affect				x	yes

^a^described as part of exposure.

^b^described as part of emotion regulation.

^c^not explicitly described as part of emotion regulation but added here, given that they are recognized emotion regulation strategies.

To be included in this review, a study had to (a) be a randomized controlled trial design, (b) evaluate a mindfulness-based mobile app, (c) assess change in one or more of our identified mechanisms using a validated, reliable measure, (d) focus on adults (≥18 years), and be (e) peer-reviewed and (f) written in English. A mindfulness-based app was defined as any app that was designed for the *sole* purpose of facilitating mindfulness practice. We excluded studies on Web-only or text-based interventions, as we were most interested in apps for their accessibility and scalability. To avoid sample biases, we also excluded studies of non-smartphone technology (e.g., VR, wearables, tablet apps), which are not yet widely adopted. We also excluded studies on adolescents because many mindfulness apps limit use to adults in their terms and conditions, and because some recent evidence suggests that mindfulness practice may affect adolescents differently than it does adults^25^. Finally, regarding validated measures, we made an exception for ecological momentary assessment (EMA) studies, which tend to use few items to reduce participant burden.

An electronic literature search was performed by the first author on October 26, 2022, on Pubmed, APA PsycINFO, and Web of Science. The search was updated on August 7, 2023. (For search strategy, see eTable 2). Studies identified were divided among four pairs of reviewers (NM & ZM, NM & TG, NM & ER, NM & SL). Reviewers independently assessed studies based on title and abstract and gave inclusion/exclusion recommendations, which were subsequently compared; any disagreements were resolved through discussion in each pair, consulting JT if consensus could not be reached. The same process was followed for full-text review, data extraction, and quality assessment (QA). The Quality Assessment Tool for Quantitative Studies, which has evidence of validity and reliability^26^, guided the quality assessment process. The tool outlines assessment criteria for eight domains of bias. Overall QA ratings and domain-specific section ratings for each study appear in eTable 3 and eTable 4, respectively.

The range of clinical and methodological characteristics in the studies included in this review prevented a meta-analysis, and we employed a narrative synthesis of the data. We first grouped studies by thematic similarity. Within each group, we assessed studies by findings, searching for similarities and differences. When findings were contradictory within a group of studies, we examined potential contributors (e.g., differences across studies in sample and study characteristics, such as control group strength, type of app evaluated, and measurement instruments). The results of this process are described in the subsequent sections.

## Results

A PRISMA flow diagram summarizing the results of our study selection process appears in eFigure 1. In total, data was collected from 5963 adults across 28 studies that varied widely in terms of location. The mean age across 23 studies that reported it was ~33 (*SD* = 8.98). Only 17 studies described the racial/ethnic composition of the sample; samples were predominantly White, and none were nationally representative. Approximately 79% identified as female (across the 24 studies that reported on female gender) and 19% as male (across the 17 studies that reported on male gender). Only one study reported on sexual orientation. See Table 2 for detailed sample characteristics.

**Table 2:** Sample characteristics.

First author & year (study location)	Sample size	MF app group (*n*)	Ctrl group (*n*)	Sample description	Age (*M*)	Age (*SD*)	Gender	Race/ethnicity	Sexual orientation
Abbott 2023 (USA)	106	50	56	Adults with elevated anxiety or worry	24	9	80% female 18% male 2% other	62% White, 14% Biracial or Multiracial, 10% Hispanic or Latinx, 8% Asian, 5% Black or AfAm, 2% NatAm	77% straight, 14% bisexual, 4% pansexual, 3% gay/lesbian, 3% asexual
Ainsworth 2022 (UK)	144	93	51	Adults with asthma	51.11	14.65	NR	MF App Group: 97% White, 3% Indian. Control Group: 93% White, 2% Chinese/SE Asian, 2% Indian, 2% NR	NR
Axelsen 2022 (Denmark)	459	167	292	Healthy adults in small- to medium-sized Danish companies	38.83	9.68	53% male 47% female	NR	NR
Bjorkstrand 2019 (Sweden)	26	11	15	Healthy university employees with high educational attainment (>12 yrs)	35.10	6.2	80% female Other genders NR	NR	NR
Gao 2022 (USA)	71	36	35	Adults withs sleep-interfering worry	41.41	17.57	80% female 17% male 3% prefer not to answer	62% White, 15% Asian, 14% Biracial or Multiracial, 7% Hispanic, 1% Black	NR
Goldberg 2020 (USA)	343	228	115	University of Wisconsin-Madison faculty, staff, and students	41.74	12.52	85% female 15% male 1% nonbinary 1% prefer not to say	82% White, 10% Multiracial, 5% Asian, 2% Black, 1% Latinx, 1% Prefer not to say	NR
Haliwa 2021 (USA)	139	69	70	College students with psychology major	19.43	1.26	81% female 19% male 1% Other	74% White, 8% Black, 6% Hispanic, 6% Asian, 1% NatAm, 5% Other	NR
Hirshberg 2022 (USA)	662	344	318	Wisconsin school system employees	<20yo: 0.2% 20–30yo: 15% 30–40yo: 29% 40–50yo: 30% 50–60yo: 21% >60yo: 4%	NR	88% female 12% male 0.1% non-binary	91% White, 5% Latinx, 4% Black/AfAm, 2% Asian/PI, 1% AI/AN	NR
Howells 2016 (11 countries)	121	57	64	“Happiness seekers” (members of self-improvement newsletters & social media groups)	40.70	10.6	87% female Other genders NR	90% White, 2% Asian/PI, 2% Hispanic, 5% Other/Multiracial, 2% Prefer not to say	NR
Huberty 2019 (USA)	72	33	39	Adults with high stress (≥14 on Perceived Stress Scale)	21.18	4.9	90% female 10% male	55% White, 17% Asian, 11% Biracial/Multiracial, 6% Black, 6% Other, 6% Prefer not to say	NR
Kubo 2019 (USA) - caregiver sample	26	13	13	Caregivers of patient sample from Kubo 2019 study	63 (median age)	NR	58% female Other genders NR	77% White, 13% Other, 6% Asian, 3% AfAm	NR
Kubo 2019 (USA) - patient sample	72	40	32	Patients with cancer currently/recently treated	59 (median age)	NR	69% female Other genders NR	65% White, 18% Other, 7% Asian, 6%AfAm, 4% Unknown	NR
Levin 2022 (USA)	16	10	13	Students on college counseling center waitlist	20.43	2.46	100% female	87% White non-Hispanic, 9% White Hispanic; 4% AI & White	NR
Low 2020 (Australia)	23	12	11	Adults with subclinical and clinical insomnia	36.39	11.74	13% male	NR	NR
Ly 2014 (Sweden)	81	41	40	Adults diagnosed with MDD	36.10	10.8	70% female 30% male	NR	NR
Orosa-Duarte 2021 (Spain)	84	31	53	Students of health sciences (medicine, psychology, nursing, or nutrition)	23	4.16	85% female NR for other genders	NR	NR
Rich 2021 (UK)	100	45	56	University employees	NR	NR	70% female 30% male	NR	NR
Roy 2021 (USA)	61	28	33	Adults with at least moderate worry (≥10 on GAD-7)	41.95	15.43	90% female 8% male 2% Other	87% White, 3% Black, 2% Asian, 8% Other	NR
Sala 2021 (USA)	228	93	135	Adults who smoke 5+ cigarettes a day & had some motivation to quit	41.48	12.48	75% female Other genders NR	81% White, 10% AfAm, 4% Hispanic/Latinx, 2% Multiracial, 1% Asian, 1% NatAm, & 1% Unknown	NR
Schulte-Frankenfeld 2021 (Germany)	64	30	34	College students who work part time	24.75	5.42	64% female 36% male	NR	NR
Sun 2021 (China)	168	84	84	Depressed pregnant women (>9 on EPDS or >4 on PHQ-9)	29.91	4.01	100% female	100% Asian (99% Han, 1% Hui)	NR
Taylor 2022 (UK)	2182	1095	1087	Adult health care workers in England	40.53	10.97	83% female 16% male 1% transgender male, other, or prefer not to say	93% White, 4% Asian, 2% Mixed or Multiracial, 1% Black	NR
van Emmerik 2018 (Netherlands)	377	191	186	Adults with an interest in mindfulness and spirituality	44.72	9.83	96% female 4% male	NR	NR
Versluis 2018 (Netherlands)	128	46	90	Adults with work stress	43.23	11.39	74% female Others NR	NR (95% Dutch)	NR
Versluis 2020 (Netherlands)	22	9	13	High-worrying young adults (45+ on PSWQ)	25.36	5.22	68% female Other genders NR	NR	NR
Walsh 2019 (Canada)	86	45	41	College students	20.02	2.53	84% female NR for the rest	NR	NR
Yang 2018 (USA)	88	45	43	All students from a Southeastern US medical school	25.11	NR	64% female 36% male	47% White, 25% Asian/PI, 10% Biracial/Multiracial, 7% Black, 6% Latinx, 6% Other	NR
Ziegler 2019 (USA)	40	22	18	Healthy young adults	18–35 (*M*NR)	NR	NR	NR	NR

*AfAm* African American, *AI/AN* American Indian Alaskan Native, *NatAm* Native American, *PI* Pacific Islander, *SE* Southeast, *NR* Not Reported.

### Study characteristics

Studies assessed Headspace (*n* = 12), VGZ Mindfulness Coach (*n* = 3), Unwinding Anxiety (*n* = 2), Healthy Minds Program (*n* = 2), Calm (*n* = 1), Stop, Breathe & Think (*n* = 1), Craving to Quit (*n* = 1), MediTrain (*n* = 1), Balloon App (*n* = 1), REM Volver a Casa (*n* = 1), Spirits Healing (*n* = 1), Wildflowers (*n* = 1), and Mindfulness (*n* = 1). These apps are available on both Apple and Android phones, except two: one offered on iPhones only (Mindfulness app^19^) and one that was commercially available at the time of investigation but now appears to be defunct (Wildflowers app^27^). (For more details on these apps, see eTable 7).

Most studies prescribed a specific dose, or amount, of app-delivered mindfulness practice (*n* = 20), ranging from 10 minutes a day (*n* = 9), several exercises a day (*n* = 5), daily (*n* = 3) or weekly (*n* = 1), or beginning at 10–20 minutes daily and gradually increasing use (*n* = 2). (For more details on app features designed to facilitate mindfulness practice, see eTable 7).

All 28 studies had at least one control group. Active control groups tended to be digital in nature, with most involving non-mindfulness apps (*n* = 10), one offering a WeChat-based health consultation, one a multimedia stress-related psychoeducation website, and one in-person MBSR. Non-mindfulness apps used to control for cognitive expectancies and attention included emotion self-monitoring apps (*n* = 3), cognitive training apps such as the 2048 app and the Peak app (*n* = 2), apps delivering other psychological interventions such as behavioral activation and progressive muscle relaxation (*n* = 2), a list-making app (*n* = 1), a music app (*n* = 1), and directions to split time equally among three apps (i.e., Duolingo, Tai Chi app, or logic games) identified in a prior study as matched in cognitive outcome expectancy (*n* = 1). Passive control group participants were either waitlisted (*n* = 15), offered treatment as usual (*n* = 2), or provided with no intervention (*n* = 1). See Table 3.

**Table 3:** Study characteristics.

First author & year (study location)	Mindfulness (MF) app tested	MF app platform^a^	Active control group	Passive control group	Type of active control group	Type of passive control group^b^	Support^c^	Incentives^d^	Dropout rate	Intervention period (weeks)	Follow-up time point (weeks)
Abbott 2023 (USA)	Headspace	Both	-	Y	-	WL	0	1	35%	4	-
Ainsworth 2022 (UK)	Headspace	Both	-	Y	-	WL	0	0	30%	6	12
Axelsen 2022 (Denmark)	Headspace	Both	Y	Y	Music app	No intervention	1	0	26%	4	-
Bjorkstrand 2019 (Sweden)	Headspace	Both	-	Y	-	WL	NR	2	0%	4	-
Gao 2022 (USA)	Unwinding Anxiety	Both	-	Y	-	TAU	1	2	11%	8	16
Goldberg 2020 (USA)	Healthy Minds Program	Both	-	Y	-	WL	0	2	46%	8	-
Haliwa 2021 (USA)	Headspace	Both	Y	-	Peak app (cognitive training)	-	1	2	10%	1.43	-
Hirshberg 2022 (USA)	Healthy Minds Program	Both	-	Y	-	WL	1	2	13%	4	12
Howells 2016 (11 countries)	Headspace	Both	Y		Catch Notes (list-making app)	-	1	NR	38%	1.43	-
Huberty 2019 (USA)	Calm	Both	-	Y	-	WL	1	2	19%	8	12
Kubo 2019 (USA) - *cg*	Headspace	Both	-	Y	-	WL	3	3	16%	8	-
Kubo 2019 (USA) - *pt*	Headspace	Both	-	Y	-	WL	3	3	26%	8	-
Levin 2022 (USA)	Stop, Breathe, and Think	Both	-	Y	-	WL	0	0	30%	4	-
Low 2020 (Australia)	Headspace	Both	Y	-	Headspace PMR app	-	NR	5	0%	6.85	-
Ly 2014 (Sweden)	Mindfulness	Apple	Y	-	Beh. activ. (BA) app by researchers	-	2	NR	14%	8	24
Orosa-Duarte 2021 (Spain)	REM Volver a casa	Both	Y	Y	Weekly in-person MBSR	WL	NR	4	45%	8	-
Rich 2021 (UK)	Headspace	Both	-	Y	-	WL	1	NR	19%	8	-
Roy 2021 (USA)	Unwinding Anxiety	Both	-	Y	-	TAU	3	2	1%	4	8
Sala 2021 (USA)	Craving to Quit	Both	Y	-	App w/ same look as MF app but only exp. sampling	-	1	3	27%	3.14	-
Schulte-Frankenfeld 2021 (Germany)	Balloon App	Both	-	Y	-	WL	NR	0	35%	8	-
Sun 2021 (China)	Spirits Healing	Both	Y	-	WeChat text-based consultation	-	1	1	31%	8	18
Taylor 2022 (UK)	Headspace	Both	Y	-	Moodzone (psychoed site)	-	1	0	35%	4	18
van Emmerik 2018 (The Netherlands)	VGZ Mindfulness Coach	Both	-	Y	-	WL	1	0	41%	8	20
Versluis 2018 (Netherlands)	VGZ Mindfulness Coach	Both	Y	Y	Emotion self-monitoring	WL	2	2	13%	4	-
Versluis 2020 (Netherlands)	VGZ Mindfulness Coach	Both	Y	-	Emotion self-monitoring	-	1	2	15%	4	-
Walsh 2019 (Canada)	Wildflowers app	Both	Y	-	2048 app (cognitive training)	-	1	1	20%	3	-
Yang 2018 (USA)	Headspace	Both	-	Y	-	WL	0	0	24%	4	8
Ziegler 2019 (USA)	MediTrain	Both	Y	-	Duolingo Tai Chi app logic games app	-	3	1	25%	6	-

^a^Apple, Android, or both; ^b^*WL* waitlist, *TAU* treatment as usual; ^c^Support: 0 = none offered, 1 = automated reminders to use app,

2 = human support provided, 3 = both 1 & 2, *NR* not reported; ^d^0 = none offered, 1 = financial incentives to use the app, 2 = financial incentives for self-report completion only, 3 = both 1 & 2, 4 = other incentive, *NR* not reported. *cg* caregiver sample. *pt* patient sample.

The average intervention phase lasted ~5.46 weeks (*SD* = 2.23). In all studies, participants were asked to train with the mindfulness app on their own (rather than in a controlled lab environment). Outcomes were measured with pre- and post-intervention self-report questionnaires in all studies but three. These three studies used objective behavioral tasks to measure outcomes, with one administering a gamified app remotely^28^ and two administering cognitive tasks in a lab environment^27,29^. Only 10 studies included follow-up assessments (i.e., assessments taking place at least one month after the end of the intervention period) to examine whether changes in the outcomes of interest to this review were sustained in the long term. (See Table 3).

App engagement metrics reported varied widely. Some reported engagement in terms of average number of minutes of app use (total or per day or week), average days practiced, and average number of app sessions/exercises completed (total or per day). As such, it was difficult to determine patterns of engagement across studies. To identify patterns, we grouped studies with similar metrics by intervention length and computed ratios based on the two metrics most often reported. Results indicated that engagement was generally low (see eTable 5).

### Methodological quality

Overall, study quality was rated as moderate to weak, with all studies having some concerns (see eTable 3). Most studies minimized measurement, allocation, and detection bias, as they assessed outcomes with valid and reliable measures or tasks, used appropriate allocation methods, and ensured research staff were blinded to condition. Bias tended to arise in terms of selection, attrition, and lack of attention on minimizing potential confounders. Most studies used self-referred convenience samples from one setting, and attrition rates ranged from moderate (i.e., 21%–40%) to high (i.e., >40%), with an average of 23% (SD = 13%) across studies. Most studies did not adjust for important confounders (see eTable 4 note). In addition, 12 studies were underpowered. Implementation bias was difficult to detect, as most studies did not report the percentage of participants who received the allocated intervention as it was intended (i.e., recommended dose of app use).

### Outcomes and findings

**Fig. 1:**
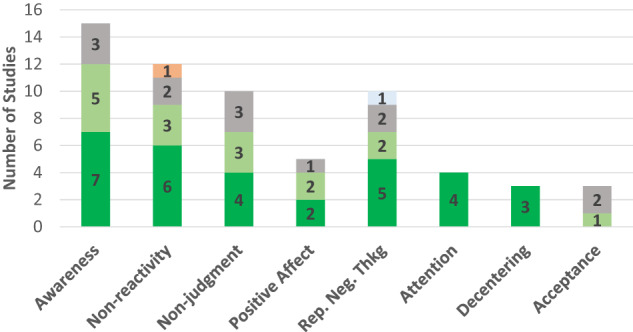
Summary of results. Dark green represents a between-group effect favoring the mindfulness app group; orange represents a between-group effect favoring the control group. Light green denotes studies that found no between-group effect (i.e., both groups improved or within-group effect favoring the mindfulness app group present); gray denotes studies that found no between-group effect (i.e., neither group improved or within-group effect favoring the control group). Light blue represents no between-group effect but unclear whether both or neither group improved. Rep. Neg. Thkg = Repetitive Negative Thinking.

Across 28 studies, 67 outcome comparisons were made between the intervention and control group. Of these 67 comparisons, 35 (53%) revealed a between-group difference favoring the intervention group. Of the 35 between-group effects favoring the intervention group, most were found when the mindfulness app was evaluated against a passive (*n* = 28; 65%) versus an active (*n* = 7; 30%) control group. (Note: Passive, or inactive, control groups involved either waitlisting participants, or offering them treatment as usual or no intervention. Active control groups offered participants a comparable task to engage in, such as a non-mindfulness app.) Effect sizes tended to be moderate to large across domains, and gains from using mindfulness apps were generally sustained at follow-up. (See Table 4). Results by outcome domain appear in Table 4 and Fig. 1.

**Table 4:** Study findings by outcome category.

T1 effect^a^	Study	MF app^b^	Results (at post-intervention)	Effect sustained at follow-up?
*Awareness (n* = *15)*				
1	Levin 2022	SBT	Medium between-group effect favoring the MF app for acting with awareness, Hedge’s *g* = 0.68 (CI −0.17, 1.58)	N/A
1	Hirshberg 2022	HMP	Small between-group effect favoring MF app for mindful action, *d* = 0.21 (CI 0.06, 0.36), *p* < 0.01	No. Trend toward significant effect at 12-week follow-up, *d* = 0.14 (CI −0.01, 0.29), *p* = 0.07
1	Rich 2021	Headspace	Medium between-group effect favoring MF app group for acting with awareness, *F*(1,122) = 8.05, *p* < 0.01, *d* = 0.51	N/A
1	Roy 2021	Unwinding Anxiety	Large between-group effect favoring MF app group for interoceptive awareness, median increase of 22 (IQR 30, *p* < 0. 01, *r* = 0.72) in MF app group & no change in controls	Yes, effect persisted at 8-week follow-up, with median increase of 26 (IQR 28.5, *p* < 0.01, *r* = 0.85) in MF app group and no sig. change in control group
1	Orosa 2021(p)	REM	Between-group effect favoring MF app group; larger changes in MF app group than controls, change = 3.6 (CI 0.1, 7.1)	N/A
1	van Emmerik 2018	VGZ Mindfulness Coach	Medium between-group effect favoring MF app group, *b* = 2.95, *SE* = 0.59, *p* < 0.01, *d* = 0.49	Yes, gains maintained at 20-week follow-up, *b* = 2.56, *SE* = 0.70, *p* < 0.01, *d* = 0.57
1	Huberty 2019	Calm	Between-group effect favoring MF app group; greater significant improvement in MF app (vs. control) group (change = 4.74, *p* < 0.01, effect size 0.83)	Yes, changes sustained at 12-week follow-up
2	Orosa 2021 (a)	REM	No between-group differences; both app and active control group improved	N/A
2	Yang 2018	Headspace	NR (only changes in primary outcomes were reported from pre- to post-intervention)	No between-group differences; both MF app and controls improved from baseline to follow-up 8 weeks later, *F*(2,138) = 4.29, *p* < 0.05
2	Kubo 2019 (pt)	Headspace	No between-group differences but trend toward significant between-group effect favoring MF app group, *F* = 3.74, *p* = 0.06, *d* = 0.43; MF app group had significant within-group increase from baseline (*M* 17.2, *SD* 3.8) to post-intervention (*M* 18.5, *SD* 3.5), *p* < 0.05	N/A
2	Ainsworth 2022	Headspace	No between-group differences but significant medium-sized increase in mindful awareness at 6 weeks in MF app group, mean diff −2.20 (CI −3.92, −0.48), *d* = 0.32	Yes, improvement of large effect size sustained at 12-week follow-up in MF app group, mean diff = −4.65 (CI −6.19, −3.10), *d* = 0.74
2	Sala 2021	Craving to Quit	No between-group differences; awareness increased in both groups, *b* = 0.01, *SE* = 0.01 (CI 0.00, 0.02), *p* < 0.05	N/A
3	Walsh 2021	Wildflowers	No between- or within-group differences	N/A
3	Haliwa 2021	Headspace	No between- or within-group differences	N/A
3	Kubo 2019 (cg)	Headspace	No between- or within-group differences	N/A
*Non-reactivity (n* = *12)*				
1	Gao 2022	Unwinding Anxiety	Significant between-group effect favoring MF app group, *β* = 3.8, *SE* = 0.78, *p* < 0.01; MF app group (control group) had a 27% (4%) average increase in non-reactivity	Yes, gains in MF group maintained at 16-week follow-up, *p* < 0.01
1	Rich 2021	Headspace	Medium between-group effect favoring MF app group for non-reactivity, *F*(1, 122) = 4.78, *p* < 0.05, *d* = 0.39	N/A
1	Roy 2021	Unwinding Anxiety	Large between-group effect favoring MF app group for non-reactivity, with median increase of 5 (IQR 6.3, *p* < 0.01, *r* = 0.95) in MF app group and no change in controls	Yes, effect persisted at 8-week follow-up, with median increase of 7.5 (IQR 6, *p* < 0.01, *r* = 0.95) in MF app group and no change in controls
1	van Emmerik 2018	VGZ	Medium between-group effect favoring MF app group for non-reactivity, *b* = 2.16, *SE* = 0.49, *p* < 0.01, *d* = 0.43	Yes, gains maintained at 20-week follow-up, *b* = 3.03, *SE* = 0.60, *p* < 0.01, *d* = 0.77
1	Huberty 2019	Calm	Between-group effect favoring MF app group; greater improvement in MF app (vs. control) group for non-reactivity (change = 3.78, *p* < 0.01, effect size 0.92)	Yes, changes sustained at 12-week follow-up
1	Orosa 2021(p)	REM	Between-group effect favoring MF app group; larger changes in MF app group than controls, change = 4.4 (CI 1.6, 7.1).	N/A
2	Orosa 2021(a)	REM	No between-group differences; both app and active control group improved	N/A
2	Yang 2018	Headspace	NR	No between-group differences; both MF app and active control group improved on non-reactivity from T1 (baseline) to T3 (follow-up 8 weeks later), *F*(2,138) = 11.45, *p* < 0.01
2	Kubo 2019 (pt)	Headspace	No between-group differences in non-reactivity but trend toward effect favoring the MF app group, *F* = 2.94, *p* = 0.09, *d* = 0.45; MF app group had a significant within-group increase from baseline (*M* 14.9, *SD* 3.7) to post-intervention (*M* 16.6, *SD* 3.3), *p* < .05.	N/A
3	Kubo 2019 (cg)	Headspace	No between-group differences in non-judgment; controls had within-group improvement from baseline (*M* 17.1, *SD* 4.2) to post-intervention (*M* 19.2, *SD* 5.1), *p* < 0.05	
3	Haliwa 2021	Headspace	No between- or within-group differences in non-reactivity	N/A
0	Levin 2022	SBT	Small between-group effect favoring the control group for non-reactivity, Hedge’s *g* = −0.31 (CI −1.17, 0.54)	N/A
*Non-judgment (n* = *10)*				
1	Levin 2022	SBT	Medium between-group effect favoring MF app group for non-judgment, Hedge’s *g* = 0.56 (CI −0.28, 1.46)	N/A
1	Orosa 2021(p)	REM	Between-group effect favoring MF app group; larger changes in MF app (vs. control) group, change = 5.7 (CI 2.2, 9.2)	N/A
1	van Emmerik 2018	VGZ	Small-to-medium between-group effect favoring MF app group, *b* = 2.19, *SE* = 0.71, *p* < 0.01, *d* = 0.34	Yes, gains maintained at 20-week follow-up, *b* = 2.68, *SE* = 0.76, *p* < 0.01, *d* = 0.47
1	Huberty 2019	Calm	Between-group effect favoring MF app group; greater improvement in MF app (vs. control) group for non-judgment, change = 4.94, *p* < 0.01, effect size 0.76	Yes, changes sustained at 12-week follow-up
2	Orosa 2021(a)	REM	No between-group differences; both groups improved	N/A
2	Kubo 2019 (pt’s)	Headspace	No between-group differences in non-judgment; MF app group had a significant within-group increase from baseline (*M* 17.3, *SD* 4.9) to post-intervention (*M* 18.4, *SD* 4.2), *p* < 0.05, but no change in controls	N/A
2	Haliwa 2021	Headspace	No between-group differences; significant increase in both groups, *F*(1,137) = 8.57, *p* < 0.01, np2 = 0.06	N/A
3	Rich 2021	Headspace	No between-group differences; trend toward significant btn-group effect favoring MF app group, *F*(1,122) = 3.32, *p* = 0.07, *d* = 0.33. (Note: Significant btn-grp effect for MF app group for completers of Headspace foundation course)	N/A
3	Yang 2018	Headspace	NR	No between-group differences; trend toward significant increase in both MF app and controls from baseline to follow-up 8 weeks later, *F*(2,140) = 2.83, *p* = 0.06
3	Kubo 2019 (cg)	Headspace	No between- or within-group differences	N/A
*Positive affect (n* = *5)*				
1	Sun 2021	Spirits Healing	Medium between-group effect favoring the MF app group for positive affect, as indicated by significant group by time interaction, *x*^2^_4 = 8.4, *p* < 0.05.	N/A
1	Howells 2016	Headspace	Medium between-group effect favoring the MF app group for positive affect, *F* = 9.13, *p* < 0.01, np2 = 0.07	N/A
2	Haliwa 2021	Headspace	No between-group differences in positive affect; significant increase in both MF app group, *F*(9,129) = 4.65, *p* < 0.01, np2 = 0.33, and control group, *F*(9,129) = 3.60, *p* < 0.01, np2 = 0.20	N/A
2	Low 2020	Headspace	No between-group differences; both groups improved on daytime positive affect, *F*(1,21) = 5.84, *p* < 0.05	
3	Versluis 2020	VGZ	No between- or within-group differences	N/A
*Repetitive negative thinking: worry (n* = *7)*				
1	Taylor 2022	Headspace	Small between-group effect favoring MF app group in terms of worry reduction, *b* = −0.30, *SE* = 0.11 (CI −0.51, −0.09), *p* < 0.01	Between-group differences were significant at 4.5 months
1	Gao 2022	Unwinding Anxiety	Significant between-group effect favoring MF app group for worry, *β* = -6.4, *SE* = 1.89, *p* < 0.01; MF app (control) group had an average worry reduction of 12% (0.3%)	Yes, gains in MF group were maintained at 16-week follow-up, *p* < 0.01
1	Roy 2021	Unwinding Anxiety	Medium-to-large between-group effect favoring the MF app group for worry, with median reduction of 7.5 (IQR 8.5, *p* < 0.01, *r* = 0.67) in MF app group but of 3 (IQR 4, *p* = 0.01, *r* = 0.44) in control grp. Mediation analysis revealed that worry reduction partially mediated the relationship between mindfulness training and anxiety reduction at 2 months, indirect effect = −0.19 (CI 0.40, −0.02), *p* < 0.05	Yes, effect persisted at 8-week follow-up, with median reduction of 15 (IQR 14.3, *p* < 0.01, *r* = 0.88) in MF app group and of 3 (IQR 6, *p* < 0.01, *r* = 0.61) in control group
2	Versluis 2018 (a)	VGZ	No between-group differences in trait worry, which decreased over time for all participants, *B* = −1.18, *p* < 0.05	N/A
2	Versluis 2018 (p)	VGZ	No between-group differences in trait worry, which decreased over time for all participants, *B* = −1.18, *p* < 0.05	N/A
3	Versluis 2020	VGZ	No between- or within-group differences	N/A
3	Abbott 2023	Headspace	No between- or within-group differences at 4 weeks	N/A
*Repetitive negative thinking: perseverative thinking (n* = *2)*				
1	Hirshberg 2022	HMP	Small-to-medium between-group effect favoring MF app, *d* = −0.35 (CI −0.51, −0.20), *p* < 0.01	Yes, persisted at 3-month follow-up, *d* = −0.22 (CI −0.37, −0.07), *p* < 0.05
1	Goldberg 2020	HMP	MF app group (vs controls) showed greater improvements in perseverative thinking, ddiff = −0.18, *p* = 0.01	N/A
*Repetitive negative thinking: rumination (n* = *1)*				
4	Taylor 2022	Headspace	No between-group differences in rumination but trend toward significant effect favoring the MF app group, *b* = −0.06, *SE* = 0.03 (CI −0.12 to 0), *p* = 0.06	No
*Attention regulation (n* = *4)*				
1	Walsh 2019	Wildflowers	Small-to-medium between-group effect favoring the MF app group for the conflict monitoring component of attentional control, estimate = −0.47 (0.21), *t*(84) = −2.29, *p* < 0.05, effect size = −0.24; no between- or within-group changes in alerting or orienting for either group	N/A
1	Axelsen 2022 (a)	Headspace	Large between-group effect favoring MF app group for sustained attention, *F*(2, 459) = 17.97, *p* < 0.01; greater significant changes in MF app group, paired *t*(166) = −10.37, *p* < 0.01, *d* = −0.80, than in active control group, paired *t*(151) = −3.62, *p* < 0.01, *d* = −0.30	N/A
1	Axelsen 2022 (p)	Headspace	Large between-group effect favoring MF app for sustained attention, *F*(2,459) = 17.97, *p* < 0.01; greater significant changes in MF app group, paired *t*(166) = −10.37, *p* < 0.01, *d* = −0.80, but none in passive control group	N/A
1	Ziegler 2019	MediTrain	Medium-to-large between-group effect favoring MF app group in sustained attention, *F*(1,37) = 6.4 (CI −17.8, −2.0), *p* < 0.05, *d* = −0.66	N/A
*Decentering/defusion (n* = *3)*				
1	Hirshberg 2022	HMP	Medium between-group effect favoring MF app, *d* = 0.40 (CI 0.25, 0.56), *p* < 0.01	Yes, persisted at 3-month follow-up, *d* = 0.35 (CI 0.20, 0.50), *p* < 0.01
1	Haliwa 2021	Headspace	Large effect favoring the MF app group for decentering, *F*(9,129) = 7.99, *p* < 0.01, np2 = 0.36	N/A
1	Goldberg 2020	HMP	MF app (versus control) group showed greater increases in defusion, ddiff = 0.41, *p* < 0.01	N/A
*Acceptance/psychological flexibility (n* = *3)*				
2	Ly 2014	MF app dev. by res group	No significant between-group differences; medium-to-large within-group differences for both MF app group, *d* = 1.06 (CI −2.33 to 4.44), *p* < 0.05, and controls, *d* = 0.80 (CI −1.61, 3.21), *p* < 0.01	Only MF app group sustained improvement at 6-month follow-up, *d* = 1.68 (CI −1.42, 4.78), *p* < 0.01
3	Ainsworth 2022	Headspace	No between- or within-group differences	No between- or within-group differences in mindful acceptance at 12-week follow-up
3	Versluis 2020	VGZ	No between- or within-group differences	N/A
*Reappraisal, suppression, self-regulation, values, & extinction (n* = *5)*				
1	Schulte 2021	Balloon App	Large between-group effect favoring MF app group for reappraisal, *F* = 9.72, *p* < 0.01, np2 = 0.14	N/A
1	Schulte 2021	Balloon App	Large between-group effect favoring MF app group for self-regulation, *F* = 15.05, *p* < 0.01, np2 = 0.20	N/A
1	Levin 2022	SBT	Large between-group effect favoring MF app for values progress, Hedge’s *g* = 0.85 (CI −0.06, 1.83)	N/A
1	Bjorkstrand 2019	Headspace	MF app group had greater significant retention of extinction learning compared to control group, as indicated by less spontaneous recovery of conditioned threat responses in the 24 h after extinction training, *t* = 2.47, *p* < 0.05, *d* = 0.98	N/A
2	Schulte 2021	Balloon App	No between-group differences in suppression; both groups improved, *F* = 5.71, *p* < 0.05, np2 = 0.08	N/A

Note. a. T1 Effect = Effect at post-intervention. MF app group = Mindfulness app group. 0 = between-group effect favoring the control group, 1 = between-group effect favoring the MF app group, 2 = no between-group differences as both groups improved or there was a within-group difference favoring MF app group, 3 = no between-group differences as neither group improved or there was a within-group difference favoring control group, 4 = no between-group differences and unclear whether both or neither improved. b. SBT = Stop, Breathe, & Think, HMP = Healthy Minds Program, REM = REM Volver a casa, VGZ = VGZ Mindfulness Coach. *Waitlist control participants received the intervention at the end of the 4-week intervention period. (a) = comparison with active control group. (p) = comparison with passive control group. (pt) patient sample. (cg) caregiver sample.

**Awareness**. The most frequently examined outcome was awareness, assessed in 15 comparisons and measured with the Acting With Awareness subscale of the Five Facet Mindfulness Questionnaire (FFMQ^30^)^31–36^ or of its short-form version (FFMQ-SF^37^)^38–40^, a one-item measure based on the FFMQ Acting With Awareness subscale in an experience sampling study^41^, the Acceptance subscale of the Philadelphia Mindfulness Scale (PHLMS^42^)^43^, the Multidimensional Assessment of Interoceptive Awareness (MAIA^44^)^45^, or the Interoceptive Respiration Task^27^. Findings were mixed, with about half the studies (*n* = 7) finding an effect favoring the intervention group (small to large effect sizes), five finding that both groups improved, and three that neither improved. Studies that found an effect favoring the intervention (versus those that did not) used passive control groups and tended to have samples with a greater female composition (see eTable 6). The four studies that used active control groups found that either both groups improved^39,41^ or neither did^27,36^.

**Nonreactivity** was assessed in 12 comparisons and measured with the nonreactivity subscale of the Five Facet Mindfulness Questionnaire (FFMQ^31^) in all but two studies that instead used the nonreactivity subscale from its 24-item short-form version (FFMQ-SF^37^)^38,40^. Findings were mixed, with six comparisons yielding an effect favoring the mindfulness app (medium to large effect sizes)^34,35,38,39,45,46^, three showing that both groups improved^33,39,40^, two that neither did^36,40^, and one yielding an effect favoring the control group^30^. All six comparisons that yielded an effect favoring the mindfulness app were made with passive control groups and tended to have samples with a greater female composition. Two studies that used active control groups found that either both groups improved^39^ or neither did^36^. The study finding an effect favoring the control group had a very small sample size and was underpowered^30^. No consistent associations between intervention length and outcomes were apparent across studies.

**Non-judgment** was assessed in 10 comparisons, using the non-judging of inner experience subscale from either the Five Facet Mindfulness Questionnaire (FFMQ^31^) or its short-form version (FFMQ-SF^37^). Findings were mixed, with four finding an effect favoring the mindfulness app^30,34,35,39^, three that both groups improved^36,39,40^, and three that neither improved^33,40,47^. Only two studies used active control groups, both finding that both groups improved^36,39^.

**Positive affect** was examined in five studies and measured with the Positive and Negative Affect Scale^48,49^ or one-item measures in EMA studies^36,50,51^. Findings were mixed, with two finding an effect favoring the intervention group^48,49^, two that both groups improved^36,51^, and one that neither group improved.^52^ All five studies used an active control group, although in two, control groups were non-equivalent^48,52^. Two of the three that found no between-group differences were underpowered^51,52^, and in one, the intervention app dose varied across participants, with some receiving it for 40 days and some for 60^51^. The two studies that found a between-group difference had samples with a greater female composition.

**Repetitive negative thinking**. Ten comparisons assessed repetitive negative thinking styles, including worry (*n* = 7), perseverative thinking (*n* = 2), and rumination (*n* = 1). Worry was assessed with the Penn State Worry Questionnaire. Three studies found an effect favoring the intervention group, with small to large effect sizes^45,46,53^, and one of these had an active control group^53^. Two studies that found that neither group improved were underpowered^52,54^. Studies that found a between-group difference (versus none) had samples with a greater female composition.

Two studies examined perseverative thinking^32,55^, assessing it with the Perseverative Thinking Questionnaire (PTQ^56^), a measure of both worry and rumination, and using a waitlist control group. Both studies found an effect favoring the mindfulness app. Only one study examined rumination directly^53^, measuring it with the brooding subscale of the Ruminative Response Scale (RRS^57^); no significant between-group differences were found.

**Attention regulation** was evaluated in only three studies (that yielded four group comparisons) and measured with behavioral tasks, including the Centre for Research on Safe Driving-Attention Network Test (CRSD-ANT^58^)^27^, which is a validated briefer version of the Attention Network Test (ANT^59^); the validated sustained attention task Test of Variables of Attention (TOVA^60^)^29^; and a gamified sustained attention task (“Go Sushi Go”)^28^ based on the validated Sustained Attention to Response Task (SART^61^). All four yielded an effect favoring the intervention group, with effect sizes ranging from small to large. All studies used an active control group.

**Decentering/defusion** was examined in three studies. Two^32,55^ used the Drexel Defusion Scale^62^ and one^36^ the decentering subscale of the Toronto Mindfulness Scale^63^. All three found a between-group difference favoring the intervention group; one had an active control group^36^.

**Acceptance/psychological flexibility** was examined in three studies and measured with the acceptance subscale of the Philadelphia Mindfulness Scale (PHLMS^64^)^43^, or with the English^65^ or Dutch^52^ version of the Acceptance and Action Questionnaire—II (AAQ-II^66^). No between-group differences were found; one study that used an active control group of a behavioral activation app found that both groups improved^65^. Two other studies found that neither group improved^43,52^, although one was underpowered^52^.

Finally, only one study each examined **self-regulation, reappraisal, suppression, values**, and **extinction**, with one study examining the first three against a waitlist control group^67^ using the Self-Regulation Scale^68^ and the German version of the Emotion Regulation Questionnaire^69^. This study found a between-group effect favoring the app group for self-regulation and reappraisal, but not suppression. One study assessed behavioral enactment of values^30^ with the Valuing Questionnaire^70^ and used a waitlist control group; results favored the intervention over the control group. The study that examined extinction^71^ used a two-day lab-based aversive Pavlovian conditioning and extinction procedure and a waitlist control group. Results showed that after using the mindfulness app for 4 weeks, the intervention (versus waitlist control) group had greater retention of extinction learning, as demonstrated by less spontaneous recovery of conditioned threat responses one day after extinction training.

### Mediation analysis

Only two studies conducted mediation analysis with a psychological disorder as an outcome. One study found that worry partially mediated the relationship between mindfulness practice and anxiety^45^ and the other that worry fully mediated the association between mindfulness training and worry-related sleep disturbance^46^.

### Heterogeneity & certainty of evidence

The range of populations in which apps were evaluated and inconsistent app engagement likely contributed to heterogeneity in findings. Methodological quality was also a likely contributor to inconsistent findings, as quality was moderate to low across studies. In the awareness domain, for example, of studies that found no between-group differences, one was underpowered,^27^one used a single-item measure that did not correlate highly with the full measure^41^, another had a 45% dropout rate^39^, and in another, data came from only 4% of eligible patients who enrolled^43^. Such methodological weaknesses, found across domains, likely increased the heterogeneity of findings and lower confidence that the lack of effects was due to a lack of app efficacy.

Methodological weaknesses also lower the certainty of evidence in domains with more consistent findings. In most domains, when effects favoring the mindfulness apps were found, most or all were from studies with passive, rather than active, control groups. In only two domains did all studies use active control groups: positive affect and attention regulation. However, in the positive affect domain, studies finding an effect favoring the mindfulness app group had relatively high attrition rates (38% and 35%), lowering confidence in findings. (For context, the average attrition rate in a recent meta-analysis of mHealth studies was 24%;^72^ objectively, attrition rates of up to 20% are considered ideal, and those nearing 40% are deemed to be high as they risk introducing bias^26^).

The domain of attention regulation was the strongest set of studies. All studies in this domain employed not just an active digital control group but also objective task measures to assess outcomes, increasing the certainty of evidence, although more studies are needed in this domain.

## Discussion

This systematic review identified 28 RCTs that evaluated a mindfulness app and examined as an outcome at least one theoretically and empirically supported mechanism of mindfulness practice. By focusing on mechanisms, this review aimed to provide a more nuanced understanding of the psychological impact of mindfulness apps. Overall, more research is needed in most outcome domains assessed in this review. Effects tended to favor the mindfulness app (versus control) group in the domains of attention regulation, repetitive negative thinking, and decentering/defusion, and findings were mixed in the domains of awareness, nonreactivity, non-judgment, positive affect, and acceptance/psychological flexibility. Various methodological issues, population characteristics, and app engagement problems likely contributed to the heterogeneity of findings.

The attention regulation domain was the strongest set of research studies. Results favoring the mindfulness app group in this domain are promising and consistent with other findings suggesting that in-person MBIs have positive effects on executive function^73,74^. They are also consistent with other study findings suggesting that those with (versus without) meditation experience exhibit greater cognitive flexibility^75^.

A trend that became apparent across most sets of studies is that studies with more female participants tended to more consistently find effects favoring the mindfulness app group. This trend is in line with other recent findings suggesting that females (versus males) may benefit more from mindfulness-based interventions^14,76–78^. Some have suggested that this difference may be due to the fact that mindfulness targets rumination, a problematic emotion regulation strategy more often used by females than males; in contrast, men tend to more often use distraction, and the focus on the present-moment experience that mindfulness training requires may initially increase negative affect for men^76^. Based on this finding, more research into these potential gender differences is warranted. If this finding is indeed replicated, gender-specific modifications in app delivery for males (e.g., emphasis on non-judgmental observation of experience) may be beneficial.

Another likely moderator of mixed findings was app engagement. Engagement metrics reported across studies varied widely, and it was difficult to assess overall engagement across the majority of studies. From the available metrics, however, engagement appeared to be generally low. The lack of consensus on engagement metrics is a recognized challenge in the mHealth space^79,80^, as is the difficulty sustaining engagement over time^81^. Notably, some studies that found no between-group differences found a mindfulness app effect at higher engagement rates^38,40^. Such findings are in line with evidence of a dose-response relationship between home practice and outcomes in in-person MBIs, which also demonstrate problems with adherence to at-home mindfulness practice, as data suggests that MBI participants complete, on average, only about 64% of the assigned amount of home practice^5^. This nevertheless amounts to a much higher rate of daily practice than seen in the studies of mindfulness apps in this review, underscoring the importance of incorporating strategies to increase app engagement so that the efficacy of these apps can be better evaluated.

It is also worth noting two other potential contributors to heterogeneity that relate to broader issues in the field. There is a lack of consensus on the definition of mindfulness, and the resulting diverse mindfulness conceptualizations^82^ may lead to different teams emphasizing different aspects of mindfulness practice during intervention implementation—differences that may have contributed to heterogeneity in outcomes. In addition, despite more mechanism-driven research into in-person MBIs over the past decade, these mechanisms are not yet well understood^83^, with some leading mindfulness mechanism theories at times yielding mixed support^84^. A better understanding of the transdiagnostic factors through which in-person MBIs impact change in mental health outcomes will lead not just to more refined mHealth interventions but also stronger evidence for the theories informing these interventions.

## Limitations of body of evidence and future directions

To advance this literature, we propose several future directions and research recommendations. First, future studies replicating these findings should employ strategies that foster app engagement. Sustained app engagement is key to obtaining accurate estimates of apps’ impact on various outcomes. In addition, although the use of incentives is acceptable in (and in line with the goals of) earlier stages of research, it is not a scalable strategy for real-world dissemination. Selecting theory-based strategies (e.g., goal-setting features, support) and building them into an app’s design, even in earlier stages of research, paves the way toward creating efficacious apps that have a greater likelihood of successful dissemination.

Related, more fine-grained details on app engagement would likely aid in resolving some of the inconsistent findings. Even mindfulness apps have a variety of features, some of which do not necessarily strengthen practice (e.g., soothing sounds or music that several apps offered, as seen in eTable 7). Better understanding how participants were using apps could help clarify why app use, in some cases, was less impactful. In addition, some people stop engaging with apps as they achieve their mental health goals, a phenomenon referred to in the literature as “e-attainment.”^85^ Thus, in some cases, discontinuation could be associated with positive outcomes, as some may have stopped using the app because mindfulness practice became a part of their routines. Thus, assessing reasons for app discontinuation can also help clarify inconsistent outcomes.

Second, future studies should better control for digital placebo effects. Many of the studies that found app effects used passive control groups, which provides encouraging evidence but does not rule out the possibility that improvements were due to simply using an app rather than to the mindfulness-specific aspects of the app. At the same time, active control groups should be chosen with careful consideration. For example, one study used a progressive muscle relaxation app as an active control and found no between-group differences in positive affect^51^. This finding may be expected, however, as relaxation has also been found to increase positive affect^86^.

Third, future studies should carefully consider the measurement of mindfulness-related constructs. There is growing concern that the conceptualization of mindfulness—and thus its measurement—is culturally biased, with some evidence suggesting that such widely used measures as the FFMQ may not actually perform well in non-Western populations^87^. Without this awareness, researchers risk continuing to build a body of evidence based on mindfulness definitions that are not necessarily universally accessible. Fortunately, alternative, more culturally relevant measures are starting to be developed^88^. In addition, although objective outcome measures are often not widely available, when they are, they should be used in future studies. Some examples of objective outcome measures include app-based cognitive games that are gamified versions of validated neuropsychological paradigms^28^, implicit tasks (e.g., the IPANAT for positive affect^89^), wearables to measure physiological reactivity (which, when combined with self-reported arousal, can be a measure of experiential avoidance^90^), or rumination induction tasks^91^ to assess whether participants who have been practicing mindfulness more are better able to exit such repetitive negative thinking states. Confidence in findings from self-report measures can be strengthened by the addition of objective measures.

With respect to study population, future studies should evaluate apps in nationally representative samples to increase the generalizability of findings. However, studies should also continue to evaluate apps in specific populations but test population-specific, theory-driven hypotheses about specific mechanisms most pertinent to that population. Doing so can help inform ways to tailor app delivery to each population to better target mechanisms. Related, greater empirical focus is needed on evaluating mindfulness apps in minoritized populations, who continue to be underrepresented in mHealth research^92^—a trend that also became apparent in the studies included in this review. Some evidence suggests that being African American is associated with lower odds of accessing and continuing to use a leading commercially available mindfulness app^93^, and lower educational attainment is also associated with lower odds of app access^93^. It is critical that future research studies focus on minoritized populations to avoid perpetuating disparities and introducing new ones in the form of digital inequities.

In addition, most studies did not report on implementation details, including details on how mindfulness was explained to participants. Yet how an intervention is introduced affects engagement and outcomes^94,95^, and calls have been made for mindfulness intervention studies to report on the explicit instruction given to participants regarding mindfulness^82^. This is especially important, given evidence that core aspects of mindfulness practice are often misunderstood by the general public^96^ and given the different conceptualizations of mindfulness^82^ that may lead to differences in intervention design and implementation. Better reporting on instruction details may elucidate some heterogeneity in findings. Researchers can also focus on other aspects of delivery beyond instructions, such as tailoring recommendations regarding timing and practice. For example, in samples of socioeconomically disadvantaged individuals facing multiple daily stressors, special attention could be placed on creating a tailored practice schedule. This discussion would help integrate mindfulness practice into their daily routine and better relate the practice to their specific challenges (e.g., constant worry regarding financial strain). This strategy may increase app relevance to each population’s contextual factors and heighten the app’s impact on hypothesized mechanisms.

Finally, moderators should be conceptualized and measured. While heterogeneity is often viewed as a signal of low efficacy, it is, in fact, normal and expected^97^. Aside from main and mediating effects, it is also important to consider when and for whom app effects are strengthened or weakened. Population-specific moderator hypotheses can relate to technology (e.g., app features), the individual (e.g., beliefs about technology), and their context (e.g., app integration into lifestyle). Special consideration should be paid to gender differences to increase our understanding of how gender influences mindfulness app outcomes. Overall, there has been little empirical focus on individual differences in the broader MBI literature too^98^, a gap that needs to be addressed in both of these areas of research.

## Guidance for clinicians: integrating apps into care

Although this review focuses on mindfulness apps’ clinical foundation, it is important to note that evidence of efficacy is just one of the five factors clinicians need to consider when selecting apps to recommend to patients. The other four factors are described in the APA app evaluation model^99^, a framework for helping clinicians choose suitable apps: accessibility (e.g., app cost, offline features), privacy and safety (i.e., data protection), app usability, and data integration toward the therapeutic goal (e.g., can app data be easily shared with the provider?)^99^. To ease the process of evaluating these factors, clinicians can use an app database, such as mindapps.org, a constantly updated database designed to make the APA framework easily actionable for public use. Using such tools can leave clinicians empowered to integrate mindfulness apps that may improve outcomes into care.

## Limitations

Several limitations of this review are worth noting. First, we did not extend the search into gray literature, which may bias results to only published evidence. Second, despite efforts to be inclusive of mindfulness mechanisms, we neglected to include self-compassion, one mechanism that has also been theoretically and empirically supported^100^. Future research should extend the focus on this important potential intermediary outcome of mindfulness app use. Third, our review did not focus on SMS-based interventions, which are also promising digital mental health tools that can enhance the impact of MBIs^101^ and thus warrant future empirical attention. Finally, given that our research question focused on discrete mechanistic targets that theories suggest would change after the onset of mindfulness practice, we excluded studies that only reported on composite measures of mindfulness (e.g., FFMQ, MAAS). Given that these scales measured several of our constructs of interest together, they were deemed out of the scope of this review. Although this limitation was partially addressed by a recent meta-analysis on composite measures of mindfulness as an outcome of mindfulness app interventions^14^, whether included studies examined mindfulness as a mechanism was not reported. Thus, a future review on this topic may be potentially fruitful.

## Conclusion

Mindfulness-based mobile apps can not only enhance mental health treatment but also offer scalable solutions to address barriers to in-person MBI access. The literature on the psychological impact of mindfulness apps is still nascent and suggests that mindfulness-based apps are promising, especially for regulating attention, reducing repetitive negative thinking, and promoting decentering/defusion. Continuing to elucidate mindfulness apps’ impact on processes of change that account for transdiagnostic symptom reduction is crucial in optimizing app design to enhance app efficacy and truly realize the potential of these apps as viable complements to routine care.

## Supplementary Information

eSUPPLEMENT

## Data Availability

While most of the data generated during this study are included in this published article, additional data on outcome measures and app engagement metrics extracted from each study is available from the corresponding author upon request.
