# Supplementary material for: The impact of mindfulness apps on psychological processes of change: a systematic review
Source: Npj Ment Health Res. 2024 Mar 18;3:14. doi: 10.1038/s44184-023-00048-5 (PMC10955957; doi:10.1038/s44184-023-00048-5)
Supplement: Supplementary file 1 — eSUPPLEMENT [file 44184_2023_48_MOESM1_ESM.pdf]

eSUPPLEMENT

eFigure 1. Study Inclusion Flowchart

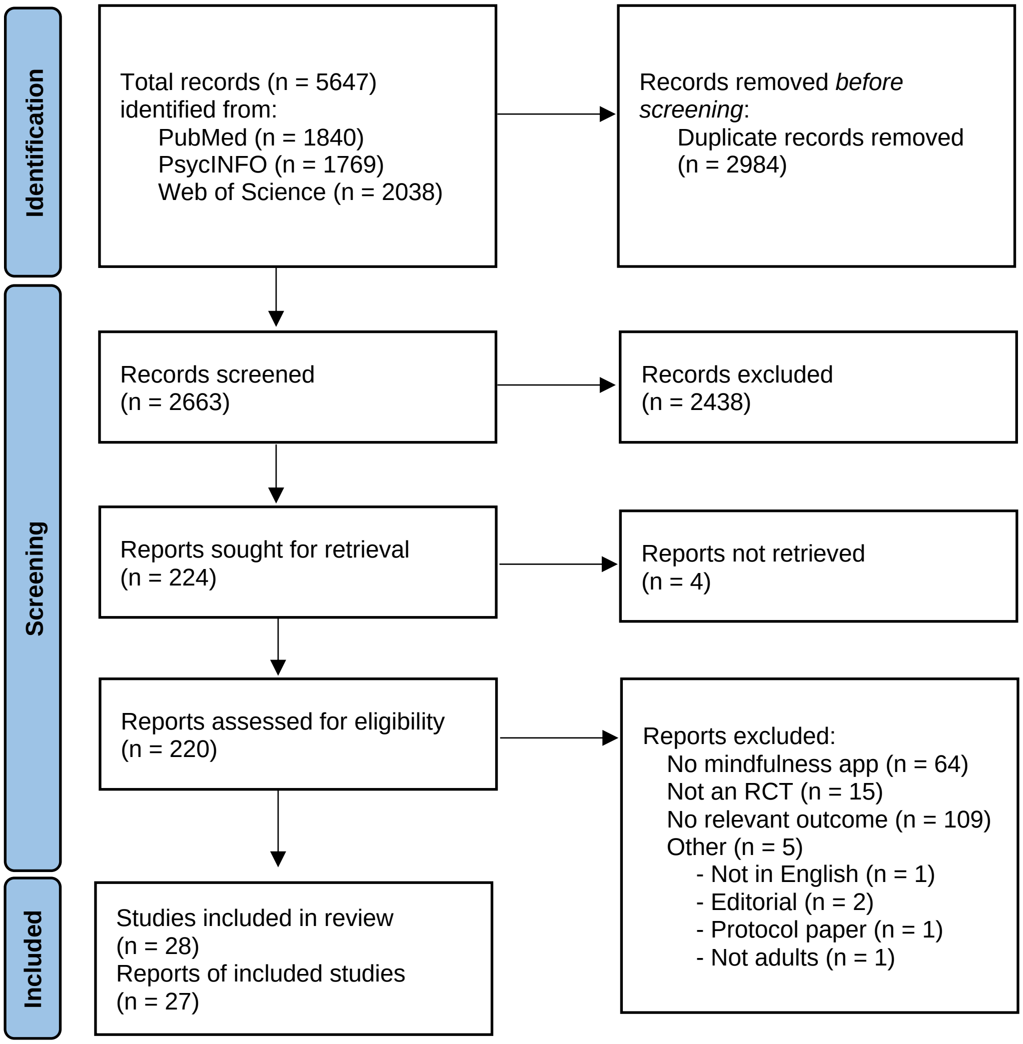

eTable1. Mindfulness Theory Papers Proposing Mechanisms of Change

Theory Paper	Mechanisms Proposed	Empirical support through mediation analysis
Shapiro et al. (2006)	Proposed a theory describing **reperceiving** as the primary mechanism of mindfulness. Reperceiving is defined as "the capacity of dispassionately observe or witness the contents of one's consciousness" (Shapiro et al., 2006). In other words, it is a change in relating to experience with greater distance so one can be more present without clinging to or pushing away experience. Reperceiving is described as "intimate detachment" and is synonymous such terms as "**decentering**," "defusion," "distancing," and "de-automatization." Shapiro and colleagues explained that change in reperceiving facilitates change in several secondary mechanisms, including **self-regulation** or self-management (reperceiving allows one to change automatic habits and access a broader range of adaptive coping skills)**, values clarification** (reperceiving allows one to understand what is meaningful to oneself rather than acting automatically based on what they have learned to be meaningful from family/society, etc.), **cognitive/emotional/behavioral flexibility** (responding rather than reflexively reacting to the environment), and **exposure** (a person can now experience even very strong emotions with greater objectivity and less reactivity).	*decentering*[1–3]<br>*self-regulation*[4]<br>*values*[4]<br>*acceptance/psychological flexibility*[2,4,5]
Holzel et al. (2011)	Proposed 4 mechanisms of action: **attention regulation, body awareness, emotion regulation** (including **reappraisal**, and **exposure**, **extinction**, and **reconsolidation**), and **change in perspective of the self**. This team of researchers suggested that these constructs are present in meditation instructions and gave a list of empirical studies in support of the idea that mindfulness meditation changes these processes. Reappraisal was defined by Holzel and colleagues (2011) as "approaching ongoing emotional reactions in a different way (**nonjudgmentally**, with **acceptance**; e.g., increases in positive reappraisal)". Exposure, extinction, and reconsolidation were, respectively, defined as "exposing oneself to whatever is present in the field of **awareness**; letting oneself be affected by it; refraining from internal reactivity (e.g., increases in **nonreactivity**)."	*attention regulation*[2,5]<br>*awareness*[2,5]<br>*emotion regulation*[6] *(re-appraisal,*[7,8]*suppression*[7,8]<br>*worry,*[7,8] *rumination*[7,8]*)*<br>*non-judgment*[5]<br>*non-reactivity*[9]
Lindsay & Creswell (2017)	Put forth the Monitor and Acceptance Theory (MAT), which suggests that **attention** monitoring and **acceptance** together mediate the effects of mindfulness practice on cognition, affect, stress, and health. The emphasis of this theory is that attentional monitoring and acceptance work synergistically to lead to the beneficial effects of mindfulness practice. Attention monitoring alone can lead to higher, rather than lower, reactivity, and thus, acceptance is key in order to relate to what one is monitoring in a non-reactive way.	*see above*
Garland, Farb, Goldin & Fredrickson (2015)	Proposed the Mindfulness-to-Meaning Theory (MMT). Rather than focusing on how mindfulness reduces negative affect and experiences, this theory centers on how it encourages positive experiences and well-being. A key mechanism connecting mindfulness practice to more distal markers of well-being is **positive affect**, brought about by the **decentering**, broadening of **attention**, and **reappraisal** that mindfulness engenders, according to MMT (Garland et al., 2015; Garland et al., 2017).	positive affect[10,11]<br>*see above for remaining constructs*

eTable2. Search Strategy

**Pubmed**<br>**Keyword Search**<br><br>Narrowed by:<br>publication year (1993-2022)	((mindful*[Title/Abstract] OR meditat*[Title/Abstract])) AND (("smartphone"[MeSH Terms] OR "smart phone*"[Title/Abstract] OR smartphone*[Title/Abstract] OR "smart phone*"[Title/Abstract] OR smart-phone*[Title/Abstract] OR cellphone*[Title/Abstract] OR "cell phone*"[Title/Abstract] OR cell-phone*[Title/Abstract] OR "cellular phone*"[Title/Abstract] OR mobile*[Title/Abstract] OR "mobile phone*"[Title/Abstract] OR "mobile device*"[Title/Abstract] OR "mobile health"[Title/Abstract] OR app[Title/Abstract] OR apps[Title/Abstract] OR application*[Title/Abstract] OR mobile-based[Title/Abstract] OR "mobile based"[Title/Abstract] OR digital[Title/Abstract] OR "digital health"[Title/Abstract] OR iphone*[Title/Abstract] OR android*[Title/Abstract] OR mhealth[Title/Abstract] OR m-health[Title/Abstract] OR tablet-based[Title/Abstract] OR ipad[Title/Abstract] OR "app delivered"[Title/Abstract] OR app-delivered[Title/Abstract]))
**APA PsycINFO**<br>**Keyword Search**<br><br>Narrowed by:<br>• publication year (1993-2022)<br>• kept academic journals, books, electronic collections<br>• excluded non-English	(AB mindful* OR TI mindful* OR AB meditat* OR TI meditat*) AND (AB smartphone* OR AB smart-phone* OR AB cellphone* OR AB "cell phone*" OR AB cell-phone* OR AB "cellular phone*" OR AB mobile* OR AB "mobile phone*" OR AB "mobile device*" OR AB "mobile health" OR AB app OR AB apps OR AB application* OR AB mobile-based OR AB "mobile based" OR AB digital OR AB "digital health" OR AB iphone* OR AB android* OR AB mhealth OR AB m-health OR AB tablet-based OR AB ipad OR AB "app delivered" OR AB app-delivered OR TI smartphone* OR TI smart-phone* OR TI cellphone* OR TI "cell phone*" OR TI cell-phone* OR TI "cellular phone*" OR TI mobile* OR TI "mobile phone*" OR TI "mobile device*" OR TI "mobile health" OR TI app OR TI apps OR TI application* OR TI mobile-based OR TI "mobile based" OR TI digital OR TI "digital health" OR TI iphone* OR TI android* OR TI mhealth OR TI m-health OR TI tablet-based OR TI ipad OR TI "app delivered" OR TI app-delivered)
**Web of Science**<br>**Keyword Search**<br><br>Narrowed by:<br>• publication year (1993-2022)<br>• kept article, early access, book chapters, review article, editorial material, letter, correction, news item<br>• excluded non-English	#3 AND (#5 OR #4)<br>#3: (((TI=(mindful*)) OR AB=(mindful*)) OR AB=(meditat*)) OR TI=(meditat*)<br>#4: (((((((((((((((((((((((TI=(smartphone*)) OR TI=(smart-phone*)) OR TI=(cellphone*)) OR TI=("cell phone*")) OR TI=(cell-phone*)) OR TI=("cellular phone*")) OR TI=(mobile*)) OR TI=("mobile phone*")) OR TI=("mobile device*")) OR TI=("mobile health")) OR TI=(app)) OR TI=(apps)) OR TI=(application*)) OR TI=(mobile-based)) OR TI=("mobile based")) OR TI=(digital)) OR TI=("digital health")) OR TI=(iphone*)) OR TI=(android*)) OR TI=(mhealth)) OR TI=(m-health)) OR TI=(tablet-based)) OR TI=(ipad)) OR TI=("app delivered")) OR TI=(app-delivered)<br>#5: (((((((((((((((((((((((AB=(smartphone*)) OR AB=(smart-phone*)) OR AB=(cellphone*)) OR AB=("cell phone*")) OR AB=(cell-phone*)) OR AB=("cellular phone*")) OR AB=(mobile*)) OR AB=("mobile phone*")) OR AB=("mobile device*")) OR AB=("mobile health")) OR AB=(app)) OR AB=(apps)) OR AB=(application*)) OR AB=(mobile-based)) OR AB=("mobile based")) OR AB=(digital)) OR AB=("digital health")) OR AB=(iphone*)) OR AB=(android*)) OR AB=(mhealth)) OR AB=(m-health)) OR AB=(tablet-based)) OR AB=(ipad)) OR AB=("app delivered")) OR AB=(app-delivered)

eTable3. Risk of Bias Ratings

	SELECTION BIAS	ALLOCA-TION BIAS	CONFOU-NDERS	BLINDING	MEASU-REMENT	ATTRITI-ON BIAS	ANALYSIS	IMPLEME-NTATION BIAS	OVERALL RATING
Abbott 2023 (USA)	W	W	W	NA	S	W	M	M	W
Ainsworth 2022 (UK)	W	S	M	NA	S	M	M	M	M
Walsh 2019 (Canada)	W	W	W	W	S	S	M	M	W
Axelsen 2022 (Denmark)	M	M	W	NA	S	M	M	M	M
Taylor 2022 (UK)	W	S	W	NA	S	M	S	M	W
Levin 2022 (USA)	W	M	M	W	S	M	M	M	W
Hirshberg 2022 (USA)	W	S	W	NA	S	S	S	M	W
Gao 2022 (USA)	W	S	M	S	S	S	S	M	M
Schulte 2021 (Germany)	W	S	W	NA	S	M	M	M	W
Sun 2021 (China)	W	S	W	S	S	M	S	M	W
Versluis 2020 (Netherlands)	W	S	W	W	S	S	M	M	W
Haliwa 2021 (USA)	W	M	W	W	W	S	S	M	W
Rich 2021 (UK)	W	M	W	W	S	S	S	M	W
Roy 2021 (USA)	W	S	W	S	S	S	S	M	W
Orosa 2021 (Spain)	W	M	W	S	S	W	M	M	W
Low 2020 (Australia)	W	W	W	W	M	S	M	M	W
Goldberg 2020 (USA)	W	S	W	NA	S	W	S	M	W
Ziegler 2019 (USA)	W	M	W	S	S	M	M	M	W
Yang 2018 (USA)	W	M	W	NA	S	M	S	M	W
van Emm. 2018 (Netherlands)	W	M	W	NA	S	W	S	M	W
Howells 2016 (11 countries)	M	M	W	NA	S	M	M	M	M
Ly 2014 (Sweden)	W	M	W	S	S	S	M	M	W
Sala 2021 (USA)	W	M	W	S	M	M	M	M	W
Bjorkstrand2019 (Sweden)	W	W	W	W	S	S	M	M	W
Huberty 2019 (USA)	W	M	W	NA	S	S	M	M	W
Kubo 2019 (USA)	M	S	W	W	S	M	M	M	W
Versluis 2018 (Netherlands)	W	S	W	W	S	S	S	M	W

Note. Risk of bias was assessed with the Quality Assessment Tool for Quantitative Studies (Thomas et al., 2004). W = Weak. M = Moderate. S = Strong. NA = Not Applicable. Tool guidance suggested an overall study rating of S if no W section ratings, M if one W section rating, and W if two or more W section ratings.

eTable4. Domain-Specific Quality Assessment Question & Section Ratings

Study	Selection		SEC	Allocation			SEC	Confounders				SEC	Blinding	SEC	Measurement		SEC	Attrition	SEC	Analysis				Notes	Implementation		
	Q1	Q2	RAT	Q1	Q2	Q3	RAT	Q1	Q2	Q3	Q4	RAT	Q1	RAT	Q1	Q2	RAT	Q1	RAT	Q1	Q2	Q3	Q4		Q1	Q2	Q3
Ainsworth 2022	SL	<60%	W	Y	Y	Y	S	Y	Y	Y	3,5,8	M	NA	NA	Y	Y	S	60-79%	M	N	N	Y	Y		NA	Y	CT
Abbott 2023	NL	NR	W	N	NR	NR	W	N	NA	Y	2-5	W	NA	NA	Y	Y	S	<60%	W	Y	N	Y	Y	underpowered	<60%	Y	CT
Walsh 2019	NL	NR	W	N	NR	N	W	N	NA	Y	2-8	W	NR	W	Y	Y	S	80-100%	S	Y	N	Y	Y	underpowered for interactions	60-79%	NR	CT
Axelsen 2022	SL	NA	M	Y	NR	N	M	CT	NA	Y	1,3-6,8	W	NA	NA	Y	Y	S	60-79%	M	Y	Y	Y	N		NR	Y	CT
Taylor 2022	SL	NR	W	Y	Y	Y	S	N	NA	Y	2,3,5-7	W	NA	NA	Y	Y	S	60-79%	M	Y	N	Y	Y		NR	Y	Y
Levin 2022	SL	NR	W	Y	Y	N	M	N	NA	Y	2,3,5	M	NR	W	Y	Y	S	60-79%	M	N	Y	Y	N	limited power	NR	NR	CT
Hirshberg 2022	NL	NA	W	Y	Y	Y	S	N	NA	Y	3,5-8	W	NA	NA	Y	Y	S	80-100%	S	Y	Y	Y	Y		NA	Y	CT
Gao 2022	NL	NA	W	Y	Y	Y	S	Y	Y	Y	2,3,5,8	W	Y	S	Y	Y	S	80-100%	S	Y	Y	Y	Y		NA	NR	CT
Schulte 2021	NL	NA	W	Y	Y	Y	S	CT	NA	Y	3,5-8	W	NA	NA	Y	Y	S	60-79%	M	Y	Y	Y	CT	potentially underpowered	NR	NR	CT
Sun 2021	SL	<60%	W	Y	Y	Y	S	N	NA	Y	3,5	W	Y	S	Y	Y	S	60-79%	M	Y	Y	Y	Y		NR	NR	CT
Versluis 2020	NL	NA	W	Y	Y	Y	S	N	NA	Y	2,3,5	W	NR	W	Y	Y	S	80-100%	S	Y	N	Y	Y	underpowered	NR	NR	CT
Haliwa 2021	NL	NR	W	Y	Y	N	M	CT	NA	Y	1-8	W	NR	W	N	N	W	80-100%	S	Y	N	Y	Y		NR	NR	CT
Rich 2021	NL	NR	W	Y	Y	N	M	N	NA	Y	3,5-7	W	NR	W	Y	Y	S	80-100%	S	Y	N	Y	Y		NR	NR	CT
Roy 2021	NL	NA	W	Y	Y	Y	S	CT	NA	Y	1-5,8	W	Y	S	Y	Y	S	80-100%	S	Y	Y	Y	Y		NR	NR	CT
Orosa 2021	NL	NR	W	Y	Y	N	M	N	NA	Y	3,5-8	W	Y	S	Y	Y	S	<60%	W	Y	N	Y	Y	underpowered	NR	NR	CT
Low 2020	NL	NA	W	N	N	N	W	CT	NA	Y	1-5,8	W	N	W	Y	Y	M	100%	S	N	N	Y	Y	underpowered	NR	NR	Y
Goldberg 2020	NL	NA	W	Y	Y	Y	S	N	NA	Y	3,5,7,8	W	NA	NA	Y	Y	S	<60%	W	Y	Y	Y	Y		NA	Y	CT
Ziegler 2019	NL	NA	W	N	NR	Y	M	CT	NA	Y	1,3-5,8	W	Y	S	Y	Y	S	60-79%	M	Y	Y	Y	CT	underpowered	NR	Y	CT
Yang 2018	SL	<60%	W	Y	Y	N	M	CT	NA	Y	2,3,5-8	W	NA	NA	Y	Y	S	60-79%	M	N	N	Y	Y		60-79%	Y	CT
van Emm. 2018	NL	NA	W	Y	Y	N	M	N	NA	Y	2,3,5-8	W	NA	NA	Y	Y	S	<60%	W	Y	Y	Y	Y		NA	Y	CT
Howells 2016	SL	NA	M	Y	Y	N	M	N	NA	Y	2,3,5-8	W	NA	NA	Y	Y	S	60-79%	M	N	Y	Y	Y		NR	Y	CT
Ly 2014	NL	NA	W	Y	Y	N	M	N	NA	Y	2,3,5,6	W	Y	S	Y	Y	S	80-100%	S	Y	N	Y	Y	underpowered	NA	NR	CT
Sala 2021	NL	NA	W	Y	NR	N	M	N	NA	Y	3,5-8	W	Y	S	Y	N	M	60-79%	M	N	N	Y	Y		NA	Y	CT
Bjork. 2019	NL	NA	W	N	NR	N	W	CT	NA	Y	1,3-5	W	NR	W	Y	Y	S	80-100%	S	Y	Y	Y	Y	underpowered	NR	NR	CT
Huberty 2019	NL	NA	W	Y	Y	N	M	N	NA	Y	3-6	W	NA	NA	Y	Y	S	80-100%	S	Y	Y	Y	N	underpowered for 2ndary outcomes	NR	Y	CT

Kubo 2019	SL	NA	M	Y	Y	Y	S	Y	N	Y	2,3,5-7	W	NR	W	Y	Y	S	60-79%	M	N	N	Y	Y	small sample	NR	Y	CT
Versluis 2018	NL	NA	W	Y	Y	Y	S	N	NA	Y	2,3,5,6	W	N	W	Y	Y	S	80-100%	S	Y	N	Y	Y		NR	NR	CT

Note. Risk of bias was assessed with the Quality Assessment Tool for Quantitative Studies (Thomas et al., 2004). Each domain of bias was assessed using the following questions, and an overall section rating was determined. **Selection:** Q1 Are the individuals selected to participate in the study likely to be representative of the target population? Q2 What percentage of selected individuals agreed to participate? **Allocation:** Q1 Is the method of random allocation stated? Q2, If the method of random allocation is stated is it appropriate? Q3 Was the method of random allocation reported as concealed? **Confounders:** Q1 Prior to the intervention were there between group differences for important confounders reported in the paper? Q2 If there were differences between groups for important confounders, were they adequately managed in the analysis? Q3 Were there important confounders not reported in the paper? Q4 List relevant confounders NOT reported in the study. **Blinding:** Q1 Was (were) the outcome assessor(s) blinded to the intervention or exposure status of participants? **Measurement:** Q1 Were data collection tools shown or are they known to be valid? Q2 Were data collection tools shown or are they known to be reliable? **Attrition:** Q1 Indicate the percentage of participants completing the study. **Analysis:** Q1 Is there a sample size calculation or power calculation? Q2 Is there a statistically significant difference between groups? Q3 Are the statistical methods appropriate? Q4 Is the analysis performed by intervention allocation status (i.e. intention to treat) rather than the actual intervention received? **Intervention Integrity:** Q1 What percentage of participants received the allocated intervention or exposure of interest? Q2 Was the consistency of the intervention measured? Q3 Is it likely that subjects received an unintended intervention (contamination or cointervention) that may influence the results? **SL** = Somewhat Likely. **NL** = Not Likely. **NA** = Not Applicable. **NR** = Not Reported. **CT** = Can't Tell. For confounders, 1 = demographic differences, 2 = prior mindfulness experience, 3 = digital literacy, 4 = education, 5 = self-efficacy, 6 = illness severity, 7 = other mental illnesses / comorbidity, 8 = currently in therapy. **SEC RAT =** Section Rating. **W** = Weak. **M** = Moderate. **S** = Strong.

eTable5. App Engagement

Metrics	Findings
We divided the average total minutes of app use in each study by the number of days of that study's intervention period to compute a more meaningful measure of minutes that could have been spent on the app across the intervention period. (Note that these daily averages were not actual averages of daily use but rather metrics calculated to make meaning of total numbers of use across different intervention periods.)	Average total minutes practiced were …<br>• 88.5 (about 9 minutes per day) in a 10-day study[12]<br>• 303 (about 14 mins/day) in a 21-day study[13]<br>• In three 4-week studies, 179 (about 6 mins/day[14]) to 89 (about 3 mins/day[15])<br>• In two 8-week studies, each reported 102 total minutes, which translates to about 2 mins of use per day[16,17]
To put the metric of average days of app use into context, we divided it by the number of available days in the intervention period in each of the 7 studies that reported this metric.	• In one 21-week study, average days practiced were 16.59 (79% of the available days[13])<br>• In four 4-week studies, average days practiced ranged from 11.97 (43%[18] to 10.88 (39%[19]) to 7.44 (27%[15]) to 3.5 (12%[20])<br>• In a 5-week study, average days practiced were 25.48 (73%[21])<br>• In one 8-week study, 10.52 (19%[16]), In a 12-week study, average days was 51 (60%[22])
The percentage of participants that adhered to the dose of the app that researchers had recommended was most commonly not reported.	Instead, some studies reported "bare minimum" use – that is, the percentage of people who used the app at least once, which ranged from 60%[18] to 70%[22] to 79%[19] to 100%.[15] Other studies reported percentages of high engagers. For example, one reported that among study completers in the intervention arm, 33% of patients and 39% of caregivers used the mindfulness app at least 70% of the days in the 8-week study period.[23] In other studies, this percentage ranged from 8% completing the entire 8-week program[24] to 53% completing the program after the 8-week intervention period and 73% at the 4-month follow-up.[25]

eTable6. Table Used in Synthesis Process

T1 Effect [A]	First Author & Year (Study Location)	MF App Tested	Active Ctrl Grp	Passive Ctrl Grp	Support [B]	Incentives [C]	Drop-out Rate	Intervention Length (wks)	F/up (wks)	MF App Grp (*N*)	Ctrl Grp (*N*)	Sample Description	Age (*M*)	Age (*SD*)	Gender	Race/ Ethnicity	Results at Post-Intervention	Effect Sustained at F/upp?
Awareness (n = 15)																		
1	Levin 2022 (USA)	Stop, Breathe, and Think	-	WL	0	0	30%	4	-	10	13	Students on college counseling center waitlist	20.43	2.46	100% female	87% White non-Hispanic, 9% White Hispanic, 4% AI & White	Medium between-group effect favoring the MF app for acting with awareness, Hedge's *g* = .68 (CI -0.17, 1.58)	N/A
1	Hirshberg 2022 (USA)	Healthy Minds Program	-	WL	1	2	13%	4	12	344	318	Wisconsin school system employees	<20y: 0.2% 20-30y: 15% 30-40y: 29% 40-50y: 30% 50-60y: 21% >60y: 4%	NR	88% female 12% male 0.1% non-binary	91% White, 5% Latinx, 4% Black/AfAm, 2% Asian/PI, 1% AI/AN	Small between-group effect favoring MF app for mindful action, *d* = 0.21 (CI 0.06, 0.36), $p < 0.01$	No. Trend toward significant effect at 12-week follow-up, *d* = 0.14 (CI −0.01, 0.29), $p = 0.07$
1	Rich 2021 (UK)	Headspace	-	WL	1	NR	19%	8	-	45	56	University employees	NR	NR	70% female 30% male	NR	Medium between-group effect favoring MF app group for acting with awareness, $F(1,122) = 8.05$, $p < 0.01$, $d = 0.51$	N/A
1	Roy 2021 (USA)	Unwinding Anxiety	-	TAU	3	2	1%	4	8	28	33	Adults with at least moderate worry (≥10 on GAD-7)	41.95	15.43	90% female 8% male 2% Other	87% White, 3% Black, 2% Asian, 8% Other	Large between-group effect favoring MF app group for interoceptive awareness, median increase of 22 (IQR 30, $p < 0.01$, $r = .72$) in MF app group & no change in controls	Yes, effect persisted at 8-week follow-up, with median increase of 26 (IQR 28.5, $p < 0.01$, $r = 0.85$) in MF app group and no sig. change in control group
1	Orosa-Duarte 2021 (Spain) (p)	REM Volver a casa	*	WL	NR	4	45%	8	-	31	53	Students of health sciences	23	4.16	85% female NR for other genders	NR	Between-group effect favoring MF app group; larger changes in MF app group than controls, change = 3.6 (CI 0.1, 7.1)	N/A
1	van Emmerik 2018 (Netherlands)	VGZ Mindfulness Coach	-	WL	1	0	41%	8	20	191	186	Adults with an interest in mindfulness & spirituality	44.72	9.83	96% female 4% male	NR	Medium between-group effect favoring MF app group, $b = 2.95$, $SE = 0.59$, $p < 0.01$, $d = 0.49$	Yes, gains maintained at 20-week follow-up, $b = 2.56$, $SE = 0.70$, $p < 0.01$, $d = 0.57$

1	Huberty 2019 (USA)	Calm	-	WL	1	2	19%	8	12	33	39	Adults with high stress (≥14 on Perceived Stress Scale)	21.18	4.9	90% female 10% male	55% White, 17% Asian, 11% Biracial or Multiracial, 6% Black, 6% Other, 6% Prefer not to say	Between-group effect favoring MF app group; greater significant improvement in MF app (vs. control) group (change = 4.74, $p < 0.01$, effect size 0.83)	Yes, changes sustained at 12-week follow-up
2	Orosa-Duarte 2021 (Spain) (a)	REM Volver a casa	Weekly in-person MBSR	*	NR	4	45%	8	-	31	53	Students of health sciences (medicine, psychology, nursing, or nutrition)	23	4.16	85% female NR for other genders	NR	No between-group differences; both app and active control group improved	N/A
2	Yang 2018 (USA)	Headspace	-	WL	0	0	24%	4	8	45	43	All students from a Southeastern US medical school	25.11	NR	64% female 36% male	47% White, 25% Asian/PI, 10% Biracial/Multiracial, 7% Black, 6% Latinx, 6% Other	NR (only changes in primary outcomes were reported from pre- to post-intervention)	No between-group differences; both MF app and controls improved from baseline to follow-up 8 weeks later, $F(2,138) = 4.29$, $p < 0.05$
2	Kubo 2019 (USA) (pt)	Headspace	-	WL	3	3	26%	8	-	40	32	Patients with cancer currently/recently treated	59 (median age)	NR	69% female Other genders NR	65% White, 18% Other, 7% Asian, 6% AfAm, 4% Unknown	No between-group differences but trend toward significant between-group effect favoring MF app group, $F = 3.74$, $p = 0.06$, $d = 0.43$; MF app group had significant within-group increase from baseline (*M* 17.2, *SD* 3.8) to post-intervention (*M* 18.5, *SD* 3.5), $p < 0.05$	N/A
2	Ainsworth 2022 (UK)	Headspace	-	WL	0	0	30%	6	12	93	51	Adults with asthma	51.11	14.65	NR	MF App Group: 97% White, 3% Indian Control Group: 93% White, 2% Chinese/SE Asian, 2% Indian, 2% NR	No between-group differences but significant medium-sized increase in mindful awareness at 6 weeks in MF app group, mean diff -2.20 (CI -3.92, -0.48), $d = .32$	Yes, improvement sustained at 12-week follow-up in MF app group, mean diff = -4.65 (CI -6.19, -3.10), $d = 0.74$
2	Sala 2021 (USA)	Craving to Quit	App w/ same look as MF but only EMA	-	1	3	27%	3.14	-	93	135	Adults who smoke 5+ cigarettes a day & had some motivation to quit	41.48	12.48	75% female Other genders NR	81% White, 10% AfAm, 4% Hispanic/Latinx, 2% Multiracial, 1% Asian, 1%	No between-group differences; awareness increased in both groups, $b = 0.01$, $SE = 0.01$ (CI 0.00, 0.02), $p < 0.05$	N/A

																NatAm, & 1% Unknown		
3	Walsh 2019 (Canada)	Wildflowers app	2048 app	-	1	1	20%	3	-	45	41	College students	20.02	2.53	84% female NR other genders	NR	No between- or within-group differences	N/A
3	Haliwa 2021 (USA)	Headspace	Peak app	-	1	2	10%	1.43	-	69	70	College students with psychology major	19.43	1.26	81% female 19% male	74% White, 8% Black, 6% Hispanic, 6% Asian, 1% NatAm, 5% Other	No between- or within-group differences	N/A
3	Kubo 2019 (USA) (cg)	Headspace	-	WL	3	3	16%	8	-	13	13	Caregivers of patient sample from Kubo 2019 study	63 (median age)	NR	58% female Other genders NR	77% White, 13% Other, 6% Asian, 3% AfAm	No between- or within-group differences	N/A
Non-reactivity (n = 12)																		
1	Gao 2022 (USA)	Unwinding Anxiety	-	TAU	1	2	11%	8	16	36	35	Adults withs sleep-interfering worry	41.41	17.57	80% female 17% male	62% White, 15% Asian, 14% Biracial or Multiracial, 7% Hispanic, 1% Black	Significant between-group effect favoring MF app group, $\beta = 3.8$, $SE = 0.78$, $p < 0.01$; MF app group (control group) had a 27% (4%) average increase in non-reactivity	Yes, gains in MF group maintained at 16-week follow-up, $p < 0.01$
1	Rich 2021 (UK)	Headspace	-	WL	1	NR	19%	8	-	45	56	University employees	NR	NR	70% female 30% male	NR	Medium between-group effect favoring MF app group for non-reactivity, $F(1,122) = 4.78$, $p < 0.05$, $d = 0.39$	N/A
1	Roy 2021 (USA)	Unwinding Anxiety	-	TAU	3	2	1%	4	8	28	33	Adults with at least moderate worry (≥10 on GAD-7)	41.95	15.43	90% female 8% male 2% Other	87% White, 3% Black, 2% Asian, 8% Other	Large between-group effect favoring MF app group for non-reactivity, with median increase of 5 (IQR 6.3, $p < 0.01$, $r = 0.95$) in MF app group and no change in controls	Yes, effect persisted at 8-week follow-up, with median increase of 7.5 (IQR 6, $p < 0.01$, $r = 0.95$) in MF app group and no change in controls
1	van Emmerik 2018 (Netherlands)	VGZ Mindfulness Coach	-	WL	1	0	41%	8	20	191	186	Adults with an interest in mindfulness & spirituality	44.72	9.83	96% female 4% male	NR	Medium between-group effect favoring MF app group for non-reactivity, $b = 2.16$, $SE = 0.49$, $p < 0.01$, $d = 0.43$	Yes, gains maintained at 20-week follow-up, $b = 3.03$, $SE = .60$, $p < 0.01$, $d = 0.77$

1	Huberty 2019 (USA)	Calm	-	WL	1	2	19%	8	12	33	39	Adults with high stress (≥14 on Perceived Stress Scale)	21.18	4.9	90% female 10% male	55% White, 17% Asian, 11% Biracial/Multiracial, 6% Black, 6% Other, 6% Prefer not to say	Between-group effect favoring MF app group; greater improvement in MF app (vs. control) group for non-reactivity (change = 3.78, $p < 0.01$, effect size 0.92)	Yes, changes sustained at 12-week follow-up
1	Orosa-Duarte 2021 (Spain) (p)	REM Volver a casa	*	WL	NR	4	45%	8	-	31	53	Students of health sciences (medicine, psychology, nursing, or nutrition)	23	4.16	85% female NR for other genders	NR	Between-group effect favoring MF app group; larger changes in MF app group than controls, change = 4.4 (CI 1.6, -7.1)	N/A
2	Orosa-Duarte 2021 (Spain) (a)	REM Volver a casa	Weekly in-person MBSR	*	NR	4	45%	8	12	31	53	Students of health sciences (medicine, psychology, nursing, or nutrition)	23	4.16	85% female NR for other genders	NR	No between-group differences; both app and active control group improved	N/A
2	Yang 2018 (USA)	Headspace	-	WL	0	0	24%	4	8	45	43	All students from a Southeastern US medical school	25.11	NR	64% female 36% male	47% White, 25% Asian/PI, 10% Biracial/Multiracial, 7% Black, 6% Latinx, 6% Other	NR	No between-group differences; both MF app and active control group improved on non-reactivity from T1 (baseline) to T3 (follow-up 8 weeks later), $F(2,138) = 11.45$, $p < 0.01$
2	Kubo 2019 (USA) (pt)	Headspace	-	WL	3	3	26%	8	-	40	32	Patients with cancer currently/recently treated	59 (median age)	NR	69% female Other genders NR	65% White, 18% Other, 7% Asian, 6% AfAm, 4% Unknown	No between-group differences in non-reactivity but trend toward effect favoring the MF app group, $F = 2.94$, $p = 0.09$, $d = 0.45$; MF app group had a significant within-group increase from baseline (M 14.9, SD 3.7) to post-intervention (M 16.6, SD 3.3), $p < 0.05$	N/A

3	Kubo 2019 (USA) (cg)	Headspace	-	WL	3	3	16%	8	-	13	13	Caregivers of patient sample from Kubo 2019 study	63 (median age)	NR	58% female Other genders NR	77% White, 13% Other, 6% Asian, 3% AfAm	No between-group differences in non-judgment; controls had within-group improvement from baseline (*M* 17.1, *SD* 4.2) to post-intervention (*M* 19.2, *SD* 5.1), $p < 0.05$	N/A
3	Haliwa 2021 (USA)	Headspace	Peak app	-	1	2	10%	1.43	-	69	70	College students with psychology major	19.43	1.26	81% female 19% male	74% White, 8% Black, 6% Hispanic, 6% Asian, 1% NatAm, 5% Other	No between- or within-group differences in non-reactivity	N/A
0	Levin 2022 (USA)	Stop, Breathe, and Think	-	WL	0	0	30%	4	-	10	13	Students on college counseling center waitlist	20.43	2.46	100% female	87% White non-Hispanic, 9% White Hispanic, 4% AI & White	Small between-group effect favoring the control group for non-reactivity, Hedge's g = -0.31 (CI -1.17, 0.54)	N/A
Non-judgment (n = 10)																		
1	Levin 2022 (USA)	Stop, Breathe, and Think	-	WL	0	0	30%	4	-	10	13	Students on college counseling center waitlist	20.43	2.46	100% female	87% White non-Hispanic, 9% White Hispanic, 4% AI & White	Medium between-group effect favoring MF app group for non-judgment, Hedge's g = .56 (CI -0.28, 1.46)	N/A
1	Orosa-Duarte 2021 (Spain) (p)	REM Volver a casa	*	WL	NR	4	45%	8	-	31	53	Students of health sciences (medicine, psychology, nursing, or nutrition)	23	4.16	85% female NR for other genders	NR	Between-group effect favoring MF app group; larger changes in MF app (vs. control) group, change = 5.7 (CI 2.2, 9.2)	N/A
1	van Emmerik 2018 (Netherlands)	VGZ Mindfulness Coach	-	WL	1	0	41%	8	20	191	186	Adults with an interest in mindfulness & spirituality	44.72	9.83	96% female 4% male	NR	Small-to-medium between-group effect favoring MF app group, $b = 2.19$, $SE = 0.71$, $p < 0.01$, $d = 0.34$	Yes, gains maintained at 20-week follow-up, $b = 2.68$, $SE = 0.76$, $p < 0.01$, $d = 0.47$
1	Huberty 2019 (USA)	Calm	-	WL	1	2	19%	8	12	33	39	Adults with high stress (≥14 on Perceived Stress Scale)	21.18	4.9	90% female 10% male	55% White, 17% Asian, 11% Biracial/Multiracial, 6% Black, 6% Other, 6% Prefer not to say	Between-group effect favoring MF app group; greater improvement in MF app (vs. control) group for non-judgment, change = 4.94, $p < 0.01$, effect size 0.76	Yes, changes sustained at 12-week follow-up

2	Orosa-Duarte 2021 (Spain) (a)	REM Volver a casa	Weekly in-person MBSR	*	NR	4	45%	8	-	31	53	Students of health sciences (medicine, psychology, nursing, or nutrition)	23	4.16	85% female NR for other genders	NR	No between-group differences; both groups improved	N/A
2	Kubo 2019 (USA) (pt)	Headspace	-	WL	3	3	26%	8	-	40	32	Patients with cancer currently/recently treated	59 (median age)	NR	69% female Other genders NR	65% White, 18% Other, 7% Asian, 6% AfAm, 4% Unknown	No between-group differences in non-judgment; MF app group had a significant within-group increase from baseline (*M* 17.3, *SD* 4.9) to post-intervention (*M* 18.4, *SD* 4.2), $p < 0.05$, but no change in controls	N/A
2	Haliwa 2021 (USA)	Headspace	Peak app	-	1	2	10%	1.43	-	69	70	College students with psychology major	19.43	1.26	81% female 19% male	74% White, 8% Black, 6% Hispanic, 6% Asian, 1% NatAm, 5% Other	No between-group differences; significant increase in both groups, $F(1,137) = 8.57$, $p < 0.01$, np2 = .06	N/A
3	Rich 2021 (UK)	Headspace	-	WL	1	NR	19%	8	-	45	56	University employees	NR	NR	70% female 30% male	NR	No between-group differences; trend toward significant between-group effect favoring MF app group, $F(1,122) = 3.32$, $p = 0.07$, $d = 0.33$. (Note: Significant between-group effect for MF app group for completers of Headspace foundation course)	N/A
3	Yang 2018 (USA)	Headspace	-	WL	0	0	24%	4	8	45	43	All students from a Southeastern US medical school	25.11	NR	64% female 36% male	47% White, 25% Asian/PI, 10% Biracial/Multiracial, 7% Black, 6% Latinx, 6% Other	NR	No between-group differences; trend toward significant increase in both MF app and controls from baseline to follow-up 8 weeks later, $F(2,140) = 2.83$, $p = 0.06$
3	Kubo 2019 (USA) (cg)	Headspace	-	WL	3	3	16%	8	-	13	13	Caregivers of patient sample from Kubo 2019 study	63 (median age)	NR	58% female Other genders NR	77% White, 13% Other, 6% Asian, 3% AfAm	No between- or within-group differences	N/A
	Positive Affect (n = 5)																	

1	Sun 2021 (China)	Spirits Healing	WeChat text-based consultation	-	1	1	31%	8	18	84	84	Depressed pregnant women (>9 on EPDS or >4 on PHQ-9)	29.91	4.01	100% female	100% Asian (99% Han, 1% Hui)	Medium between-group effect favoring the MF app group for positive affect, as indicated by significant group by time interaction, $x2\_4 = 8.4$, $p < 0.05$	N/A
1	Howells 2016 (11 countries)	Headspace	Catch Notes (list-making app)	-	1	NR	38%	1.43	-	57	64	"Happiness seekers" (members of self-improv. newsletters & soc. med. groups)	40.70	10.6	87% female Others NR	90% White, 2% Asian/PI, 2% Hispanic, 5% Other/Multiracial, 2% Prefer not to say	Medium between-group effect favoring the MF app group for positive affect, F = 9.13, $p < 0.01$, np2 = .07	N/A
2	Haliwa 2021 (USA)	Headspace	Peak app	-	1	2	10%	1.43	-	69	70	College students with psychology major	19.43	1.26	81% female 19% male	74% White, 8% Black, 6% Hispanic, 6% Asian, 1% NatAm, 5% Other	No between-group differences in positive affect; significant increase in both MF app group, $F(9,129) = 4.65$, $p < 0.01$, np2 = 0.33, and control group, $F(9,129) = 3.60$, $p < 0.01$, np2 = 0.20	N/A
2	Low 2020 (Australia)	Headspace	Headspace PMR App	-	NR	5	0%	6.85	-	12	11	Adults with subclinical and clinical insomnia	36.39	11.74	13% male	NR	No between-group differences; both groups improved on daytime positive affect, $F(1,21) = 5.84$, $p < 0.05$	
3	Versluis 2020 (Netherlands)	VGZ Mindfulness Coach	Emotion self-monitoring	-	1	2	15%	4	-	9	13	High-worrying young adults (45+ on PSWQ)	25.36	5.22	68% female Other genders NR	NR	No between- or within-group differences	N/A
Repetitive Negative Thinking: Worry (n = 7)																		
1	Taylor 2022 (UK)	Headspace	Moodzone (psychoed site)	-	1	0	35%	4	18	1095	1087	Adult health care workers in England	40.53	10.97	83% female 16% male	93% White, 4% Asian, 2% Mixed or Multiracial, 1% Black	Small between-group effect favoring MF app group in terms of worry reduction, $b = -0.30$, SE 0.11 (CI -0.51, -0.09), $p < 0.01$	Between-group differences were significant at 4.5 months
1	Gao 2022 (USA)	Unwinding Anxiety	-	TAU	1	2	11%	8	16	36	35	Adults withs sleep-interfering worry	41.41	17.57	80% female 17% male	62% White, 15% Asian, 14% Biracial or Multiracial, 7% Hispanic, 1% Black	Significant between-group effect favoring MF app group for worry, $\beta = -6.4$, $SE = 1.89$, $p < 0.01$; MF app (control) group had an average worry reduction of 12% (0.3%)	Yes, gains in MF group were maintained at 16-week follow-up, $p < 0.01$

1	Roy 2021 (USA)	Unwinding Anxiety	-	TAU	3	2	1%	4	8	28	33	Adults with at least moderate worry (≥10 on GAD-7)	41.95	15.43	90% female 8% male 2% Other	87% White, 3% Black, 2% Asian, 8% Other	Medium-to-large between-group effect favoring the MF app group for worry, with median reduction of 7.5 (IQR 8.5, $p < 0.01$, $r = 0.67$) in MF app group but of 3 (IQR 4, $p = 0.01$, $r = 0.44$) in control group. Mediation analysis revealed that worry reduction partially mediated the relationship between mindfulness training and anxiety reduction at 2 months, indirect effect = -0.19 (CI 0.40, -0.02), $p < 0.05$	Yes, effect persisted at 8-week follow-up, with median reduction of 15 (IQR 14.3, $p < 0.01$, $r = 0.88$) in MF app group and of 3 (IQR 6, $p < 0.01$, $r = 0.61$) in control group
2	Versluis 2018 (Netherlands) (a)	VGZ Mindfulness Coach	Emotion self-monitoring	*	2	2	13%	4	-	46	90	Adults with work stress	43.23	11.39	74% female Other genders NR	NR (95% Dutch)	No between-group differences in trait worry, which decreased over time for all participants, $B = -1.18$, $p < 0.05$	N/A
2	Versluis 2018 (Netherlands) (p)	VGZ Mindfulness Coach	*	WL	2	2	13%	4	-	46	90	Adults with work stress	43.23	11.39	74% female Other genders NR	NR (95% Dutch)	No between-group differences in trait worry, which decreased over time for all participants, $B = -1.18$, $p < 0.05$	N/A
3	Versluis 2020 (Netherlands)	VGZ Mindfulness Coach	Emotion self-monitoring	-	1	2	15%	4	-	9	13	High-worrying young adults (45+ on PSWQ)	25.36	5.22	68% female Other genders NR	NR	No between- or within-group differences	N/A
3	Abbott 2023 (USA)	Headspace	-	WL	0	1	35%	4	-	50	56	Adults with elevated anxiety or worry	24	9	80% female 18% male 2% other	62% White, 14% Biracial or Multiracial, 10% Hispanic or Latinx, 8% Asian, 5% Black or AfAm, 2% NatAm	No between- or within-group differences at 4 weeks	N/A
Repetitive Negative Thinking: Perseverative Thinking (n = 2)																		
1	Hirshberg 2022 (USA)	Healthy Minds Program	-	WL	1	2	13%	4	12	344	318	Wisconsin school system employees	<20y: 0.2% 20-30y: 15% 30-40y: 29% 40-50y: 30% 50-60y: 21% >60y: 4%	NR	88% female 12% male 0.1% non-binary	91% White, 5% Latinx, 4% Black/AfAm, 2% Asian/PI, 1% AI/AN	Small-to-medium between-group effect favoring MF app, $d = -0.35$ (CI -0.51, -0.20), $p < 0.01$	Yes, persisted at 3-month follow-up, $d = -0.22$ (CI -0.37, -0.07), $p < 0.05$

1	Goldberg 2020 (USA)	Healthy Minds Program	-	WL	0	2	46%	8	-	228	115	University of Wisconsin-Madison faculty, staff, and students	41.74	12.52	85% female 15% male	82% White, 10% Multiracial, 5% Asian, 2% Black, 1% Latinx, 1% Prefer not to say	MF app group (vs. controls) showed greater improvements in perseverative thinking, ddiff = -0.18, *p* = 0.01	N/A
Repetitive Negative Thinking: Rumination (n = 1)																		
4	Taylor 2022 (UK)	Headspace	Moodzone (psychoed site)	-	1	0	35%	4	18	1095	1087	Adult health care workers in England	40.53	10.97	83% female 16% male	93% White, 4% Asian, 2% Mixed or Multiracial, 1% Black	No between-group differences in rumination but trend toward significant effect favoring the MF app group, b = -0.06, SE = 0.03 (CI -0.12, 0), p = 0.06	No
Attention Regulation (n = 4)																		
1	Walsh 2019 (Canada)	Wildflowers app	2048 app	-	1	1	20%	3	-	45	41	College students	20.02	2.53	84% female NR other genders	NR	Small-to-medium between-group effect favoring the MF app group for the conflict monitoring component of attentional control, estimate = -0.47 (0.21), $t(84) = -2.29$, $p < 0.05$, effect size = -0.24; no between- or within-group changes in alerting or orienting for either group	N/A
1	Axelsen 2022 (Denmark) (a)	Headspace	Music app	*	1	0	26%	4	-	167	292	Healthy adults in small- to medium-sized Danish companies	38.83	9.68	53% male 47% female	NR	Large between-group effect favoring MF app group for sustained attention, $F(2,459) = 17.97$, $p < 0.01$; greater significant changes in MF app group, paired $t(166) = -10.37$, $p < 0.01$, $d = -0.80$, than in active control group, paired $t(151) = -3.62$, $p < 0.01$, $d = -0.30$	N/A
1	Axelsen 2022 (Denmark) (p)	Headspace	*	No intervention	1	0	26%	4	-	167	292	Healthy adults in small- to medium-sized Danish companies	38.83	9.68	53% male 47% female	NR	Large between-group effect favoring MF app group for sustained attention, $F(2,459) = 17.97$, $p < 0.01$; greater significant changes in MF app group, paired $t(166) = -10.37$, $p < 0.01$, $d = -0.80$, but none in passive control group	N/A

1	Ziegler 2019 (USA)	MediTrain	Duolingo, Tai Chi app, logic games app	-	3	1	25%	6	-	22	18	Healthy young adults	18-35 (*M* age NR)	NR	NR	NR	Medium-to-large between-group effect favoring MF app group in sustained attention, $F(1,37) = 6.4$ (CI -17.8, -2.0), $p < 0.05$, $d = -0.66$	N/A
Decentering/defusion (n = 3)																		
1	Hirshberg 2022 (USA)	Healthy Minds Program	-	WL	1	2	13%	4	12	344	318	Wisconsin school system employees	<20y: 0.2%<br>20-30y: 15%<br>30-40y: 29%<br>40-50y: 30%<br>50-60y: 21%<br>>60y: 4%	NR	88% female<br>12% male<br>0.1% non-binary	91% White, 5% Latinx, 4% Black/AfAm, 2% Asian/PI, 1% AI/AN	Medium between-group effect favoring MF app, $d = 0.40$ (CI 0.25 to 0.56), $p < 0.01$	Yes, persisted at 3-month follow-up, $d = 0.35$ (CI 0.20, 0.50), $p < 0.01$
1	Haliwa 2021 (USA)	Headspace	Peak app	-	1	2	10%	1.43	-	69	70	College students with psychology major	19.43	1.26	81% female<br>19% male	74% White, 8% Black, 6% Hispanic, 6% Asian, 1% NatAm, 5% Other	Large effect favoring the MF app group for decentering, $F(9,129) = 7.99$, $p < 0.01$, np2 = 0.36	N/A
1	Goldberg 2020 (USA)	Healthy Minds Program	-	WL	0	2	46%	8	-	228	115	University of Wisconsin-Madison faculty, staff, and students	41.74	12.52	85% female<br>15% male	82% White, 10% Multiracial, 5% Asian, 2% Black, 1% Latinx, 1% Prefer not to say	MF app (vs. control) group showed greater increases in defusion, ddiff = .41, $p < 0.01$	N/A
Acceptance / Psychological Flexibility (n = 3)																		
2	Ly 2014 (Sweden)	Mindfulness	Behavioral activation (BA) app by researchers	-	2	NR	14%	8	24	41	40	Adults diagnosed with MDD	36.10	10.8	70% female<br>30% male	NR	No significant between-group differences; medium-to-large within-group differences for both MF app group, $d = 1.06$ (CI -2.33, 4.44), $p < 0.05$, and controls, $d = 0.80$ (CI -1.61, 3.21), $p < 0.01$	Only MF app group sustained improvement at 6-month follow-up, $d = 1.68$ (CI -1.42, 4.78), $p < 0.01$
3	Ainsworth 2022 (UK)	Headspace	-	WL	0	0	30%	6	12	93	51	Adults with asthma	51.11	14.65	NR	MF App Grp: 97% White, 3% Indian<br>Ctrl Grp: 93% White, 2% Chinese/SE Asian, 2% Indian, 2% NR	No between- or within-group differences	No between- or within-group differences in mindful acceptance at 12-week follow-up
3	Versluis 2020 (Netherlands)	VGZ Mindfulness Coach	Emotion self-monitoring	-	1	2	15%	4	-	9	13	High-worrying young adults (45+ on PSWQ)	25.36	5.22	68% female<br>Other genders NR	NR	No between- or within-group differences	N/A

Reappraisal, Suppression, Self-Regulation, Values, & Extinction (n = 5)																		
1	Schulte-Frankenfeld 2021 (Germany)	Balloon App	-	WL	NR	0	35%	8	-	30	34	College students who work part time	24.75	5.42	64% female 36% male	NR	Large between-group effect favoring MF app group for reappraisal, $F = 9.72$, $p < 0.01$, np2 = 0.14	N/A
1	Schulte-Frankenfeld 2021 (Germany)	Balloon App	-	WL	NR	0	35%	8	-	30	34	College students who work part time	24.75	5.42	64% female 36% male	NR	Large between-group effect favoring MF app group for self-regulation, $F = 15.05$, $p < 0.01$, np2 = 0.20	N/A
1	Levin 2022 (USA)	Stop, Breathe, and Think	-	WL	0	0	30%	4	-	10	13	Students on college counseling center waitlist	20.43	2.46	100% female	87% White non-Hispanic, 9% White Hispanic, 4% AI & White	Large between-group effect favoring MF app for values progress, Hedge's $g = .85$ (CI -0.06, 1.83)	N/A
1	Bjorkstrand 2019 (Sweden)	Headspace	-	WL	NR	2	0%	4	-	11	15	Health university employees with high educational attainment (>12 yrs)	35.1	6.2	80% female Other genders NR	NR	MF app group had greater significant retention of extinction learning compared to control group, as indicated by less spontaneous recovery of conditioned threat responses in the 24 h after extinction training, $t = 2.47$, $p < 0.05$, $d = 0.98$	N/A
2	Schulte-Frankenfeld 2021 (Germany)	Balloon App	-	WL	NR	0	35%	8	-	30	34	College students who work part time	24.75	5.42	64% female 36% male	NR	No between-group differences in suppression; both groups improved, $F = 5.71$, $p < 0.05$, $np2 = 0.08$	N/A

Note. **Top row**: T1 Effect = Effect at post-intervention; MF app = Mindfulness app; Grp = Group; Ctrl = Control; wks = weeks; F/up = follow-up; [A]Codes for effect at post-intervention (0 = between-group effect favoring the control group, 1 = between-group effect favoring the MF app group, 2 = no between-group differences as both groups improved or there was a within-group difference favoring MF app group, 3 = no between-group differences as neither group improved or there was a within-group difference favoring control group, 4 = no between-group differences and unclear whether both or neither improved); [B]Codes for support types (0 = none offered, 1 = automated reminders to use app, 2 = human support provided, 3 = both 1 & 2, NR = Not Reported); [C]Codes for incentives (0 = none offered, 1 = financial incentives to use the app, 2 = financial incentives for self-report completion only, 3 = both 1 & 2, 4 = Other incentive, NR = Not Reported). **Demographics column**: Demographics presented here as reported in each study; AfAm = African American, AI/AN = American Indian / Alaska Native, NatAm = Native American, PI = Pacific Islander, SE = Southeast, NR = Not Reported. **First Author & Year (Study Location) column:** (a) = this row refers to data from comparison of MF app group to active control group; (p) = this row refers to data from comparison of MF app group to passive control group; (pt) = study with pt sample; (cg) = study with caregiver sample. **Control Group columns:** if * appears in Active (Passive) Ctrl Grp column = this study included an active (passive) control group but this row refers to data from comparison of MF app group with passive (active) control group. WL = Waitlist control group.

eTable 7. App Characteristics

			Key Mindfulness (MF) Content				***Primary Format***			***Supporting Features***					
**App name**	**App developer**	**Languages**	**MF basics**	**Themed practice (e.g., MF & pain)**	**Guided timed practice**	**Un-guided timed practice**	**Writ-ten**	**Audio**	**Video**	**Mood check-in**	**Other interac-tive tools**	**Msg board**	**Practice tracker**	**Practice remin-ders**	**Other features**
Headspace	Headspace Inc.	1-5	x	x	x			x	x				x	x	mindful movement, music, mental health podcasts
VGZ Mindful-ness Coach	Zorgver-zekeraar VGZ	8	x		x			x							mindful movement
Unwinding Anxiety	Mind-Sciences, Inc.	1	x	x	x		x	x	x	x	x	x	x	x	sensation check-ins & practice suggestions, worry-specific info, goal setting feature
Healthy Minds Program	Healthy Minds Innov-ations, Inc.	1	x	x	x	x		x			x		x	x	customizable duration & format, self-assessments, podcasts
Calm	Calm.com, Inc.	1-7	x	x	x			x	x	x	x		x	x	reflection prompts, sleep tracker, podcasts, music, soothing sounds
*Stop, Breathe & Think	Stop, Breathe & Think	1 *(app not avail.)*	x	x	x			x		x					check-in & practice suggestions
Craving to Quit	Sharecare, Inc.	1	x	x	x			x	x	x	x	x	x	x	Q&A with expert; working mindfully with cravings
*Medi-Train	Neuroscape Center at UCSF	1	x		x	x	x	x		x	x		x		Interactive screen assessing ability to focus on breath, daily tips, progress graph
*Balloon App	MissionMe (Gruner + Jahr Deutschland GmbH)	4 *(app avail. only in Germany)*	x	x	x			x					x	x	Calendar of practice, themed courses (e.g., Sleep Better, Reduce Stress)

REM Volver a Casa	Espacio de Formación en Salud y Psicoterapia	2	x	x	x	x		x	x	x			x	Special focus on integrating MF in daily life
*Spirits Healing	NR	10 *(app avail. only in mainland China)*	x	x	x		x	x	x		x			Mindfulness journal
*Wild-flowers app	Mobio Interactive Inc.	*1 (app not avail.)*	x	x	x		x	x		x				Practice suggestions based on mood & stress level
*Mind-fulness app	NR	*UNC (app not avail.)*	x		x	x		x						

Note. Languages: 1 = English, 2 = Spanish, 3 = French, 4 = German, 5 = Portuguese, 6 = Korean, 7 = Japanese, 9 = Dutch, 10 = Chinese, UNC = Unclear. MF basics = Mindfulness psychoeducation and instruction in basic mindfulness techniques. App not avail. = App no longer available in app stores. MF = Mindfulness. *App not accessible so features were rated based on app description in the papers that evaluated it.

References

1. Hayes-Skelton, S. A. & Lee, C. S. Decentering in Mindfulness and Cognitive Restructuring for Social Anxiety: An Experimental Study of a Potential Common Mechanism. *Behav Modif* **44**, 817–840 (2020).
2. Tran, U. S. *et al.* The Serenity of the Meditating Mind: A Cross-Cultural Psychometric Study on a Two-Factor Higher Order Structure of Mindfulness, Its Effects, and Mechanisms Related to Mental Health among Experienced Meditators. *PLOS ONE* **9**, e110192 (2014).
3. Hoge, E. A. *et al.* Change in Decentering Mediates Improvement in Anxiety in Mindfulness-Based Stress Reduction for Generalized Anxiety Disorder. *Cognit Ther Res* **39**, 228–235 (2015).
4. Carmody, J., Baer, R. A., L. B. Lykins, E. & Olendzki, N. An empirical study of the mechanisms of mindfulness in a mindfulness-based stress reduction program. *Journal of Clinical Psychology* **65**, 613–626 (2009).
5. Burzler, M. A., Voracek, M., Hos, M. & Tran, U. S. Mechanisms of Mindfulness in the General Population. *Mindfulness* **10**, 469–480 (2019).
6. Freudenthaler, L., Turba, J. D. & Tran, U. S. Emotion Regulation Mediates the Associations of Mindfulness on Symptoms of Depression and Anxiety in the General Population. *Mindfulness (N Y)* **8**, 1339–1344 (2017).
7. Parmentier, F. B. R. *et al.* Mindfulness and Symptoms of Depression and Anxiety in the General Population: The Mediating Roles of Worry, Rumination, Reappraisal and Suppression. *Frontiers in Psychology* **10**, (2019).
8. Desrosiers, A., Vine, V., Klemanski, D. H. & Nolen-Hoeksema, S. Mindfulness and emotion regulation in depression and anxiety: common and distinct mechanisms of action. *Depress Anxiety* **30**, 654–661 (2013).
9. Britton, W. B., Shahar, B., Szepsenwol, O. & Jacobs, W. J. Mindfulness-based cognitive therapy improves emotional reactivity to social stress: results from a randomized controlled trial. *Behav Ther* **43**, 365–380 (2012).
10. Batink, T., Peeters, F., Geschwind, N., van Os, J. & Wichers, M. How does MBCT for depression work? studying cognitive and affective mediation pathways. *PLoS One* **8**, e72778 (2013).
11. McLaughlin, L. E., Luberto, C. M., O'Bryan, E. M., Kraemer, K. M. & McLeish, A. C. The indirect effect of positive affect in the relationship between trait mindfulness and emotion dysregulation. *Pers Individ Dif* **145**, 70–74 (2019).
12. Haliwa, I., Ford, C. G., Wilson, J. M. & Shook, N. J. A Mixed-Method Assessment of a 10-Day Mobile Mindfulness Intervention. *Front Psychol* **12**, 722995 (2021).
13. Walsh, K., Saab, B. & Farb, N. Effects of a Mindfulness Meditation App on Subjective Well-Being: Active Randomized Controlled Trial and Experience Sampling Study. *JMIR MENTAL HEALTH* **6**, (2019).
14. Axelsen, J. L., Meline, J. S. J., Staiano, W. & Kirk, U. Mindfulness and music interventions in the workplace: assessment of sustained attention and working memory using a crowdsourcing approach. *BMC Psychol* **10**, 108 (2022).
15. Levin, M. E., Hicks, E. T. & Krafft, J. Pilot evaluation of the stop, breathe & think mindfulness app for student clients on a college counseling center waitlist. *J Am Coll Health* **70**, 165–173 (2022).
16. Goldberg, S. B. *et al.* Alliance With an Unguided Smartphone App: Validation of the Digital Working Alliance Inventory. *Assessment* **29**, 1331–1345 (2022).

17. Rich, A. *et al.* Evaluation of a novel intervention to reduce burnout in doctors-in-training using self-care and digital wellbeing strategies: a mixed-methods pilot. *BMC Med Educ* **20**, 294 (2020).
18. Yang, E., Schamber, E., Meyer, R. M. L. & Gold, J. I. Happier Healers: Randomized Controlled Trial of Mobile Mindfulness for Stress Management. *The Journal of Alternative and Complementary Medicine* **24**, 505–513 (2018).
19. Hirshberg, M. J. *et al.* A randomized controlled trial of a smartphone-based well-being training in public school system employees during the COVID-19 pandemic. *Journal of Educational Psychology* (2022) doi:10.1037/edu0000739.
20. Versluis, A., Verkuil, B., Spinhoven, P. & Brosschot, J. F. Feasibility and effectiveness of a worry-reduction training using the smartphone: A pilot randomised controlled trial. *British Journal of Guidance & Counselling* **48**, 227–239 (2020).
21. van Emmerik, A. A. P., Berings, F. & Lancee, J. Efficacy of a Mindfulness-Based Mobile Application: a Randomized Waiting-List Controlled Trial. *Mindfulness (N Y)* **9**, 187–198 (2018).
22. Ainsworth, B. *et al.* A feasibility trial of a digital mindfulness-based intervention to improve asthma-related quality of life for primary care patients with asthma. *J Behav Med* **45**, 133–147 (2022).
23. Kubo, A. *et al.* A Randomized Controlled Trial of mHealth Mindfulness Intervention for Cancer Patients and Informal Cancer Caregivers: A Feasibility Study Within an Integrated Health Care Delivery System. *Integr Cancer Ther* **18**, 153473541985063 (2019).
24. Sun, Y. *et al.* Effectiveness of Smartphone-Based Mindfulness Training on Maternal Perinatal Depression: Randomized Controlled Trial. *J Med Internet Res* **23**, e23410 (2021).
25. Gao, M. *et al.* Targeting Anxiety to Improve Sleep Disturbance: A Randomized Clinical Trial of App-Based Mindfulness Training. *Psychosom Med* **84**, 632–642 (2022).
